# Thermoelectric PANI/TeNWs Fiber Based Microsensor for Passive Temperature and Active Chemical Sensing

**DOI:** 10.1002/advs.76311

**Published:** 2026-06-29

**Authors:** Dongmei Xie, Mengran Chen, Xuefei Zhang, Yan Xu, Weiye Geng, Zhe Tang, Si‐ze Lou, Shun Wan, Zhenguo Liu, Heng Liu, Peng‐an Zong

**Affiliations:** ^1^ College of Materials Science and Engineering Nanjing Tech University Nanjing China; ^2^ State Key Laboratory of New Ceramics and Fine Processing School of Materials Science and Engineering Tsinghua University Beijing China; ^3^ Key Laboratory of Flexible Electronics of Zhejiang Province Ningbo Institute of Northwestern Polytechnical University Ningbo China; ^4^ Advanced Institute for Materials Research (WPI‐AIMR) Tohoku University Sendai Japan

**Keywords:** gas sensor, micrometer‐scale sensor, pH sensor, thermoelectric, wet‐spinning

## Abstract

Micrometer‐scale chemical sensors serve as pivotal functional components that bridge microscopic environments and macroscopic information systems, yet integrating multiple sensing modalities within a single microscale fiber without compromising performance remains a challenge in materials design. Herein, we report a continuous wet‐spinning process to fabricate multifunctional microfibers that integrate chemically responsive polyaniline (PANI) with high‐Seebeck‐coefficient tellurium nanowires (TeNWs). The optimized PANI/TeNWs composite fiber (60 wt.% TeNWs) achieves a Seebeck coefficient of 59.9 µV K^−1^ and a power factor of 9.2 µW m^−1^ K^−2^, enabling passive temperature monitoring with a detection limit of 1 K, where thermal gradients directly generate the sensing signal. Simultaneously, the fiber demonstrates remarkable chemical sensing capabilities, exhibiting a Nernstian pH response (59.25 mV pH^−1^) and rapid ammonia detection (0.96 s at 50 ppm). By demonstrating both passive thermoelectric transduction and active chemical sensing in a single microfiber platform, this work establishes a versatile material system that combines energy‐autonomous temperature sensing with high‐performance chemical detection. This integrated approach offers substantial application prospects in precision medicine and environmental safety monitoring, where miniaturized sensors with diverse functionalities are urgently needed.

## Graphical abstract

Continuous wet‐spinning of PANI with TeNWs yields a multifunctional microfiber. Oriented TeNWs impose chain alignment and enhance π‐electron delocalization, boosting the Seebeck coefficient to 59.9 µV K^−1^ for passive temperature sensing (1 K detection limit), while achieving high pH sensitivity (59.25 mV pH^−1^) and rapid NH_3_ response (0.96 s). This design establishes an integrable micro‐platform for next‐generation multimodal sensors.

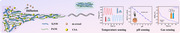

## 1 Introduction

Research in microsystems, wearable electronics, and implantable medical devices is advancing toward miniaturized and integrated sensing platforms with low power consumption and higher functional density [1, 2, 3]. However, conventional sensors are typically constructed from discrete sensing components, rigid supports, and external power modules, with dimensions ranging from millimeters to centimeters [4, 5]. Such macroscopic architectures limit spatial resolution and localized in situ detection, require relatively large sample volumes, and may disturb sensitive microenvironments through contact stress [6, 7]. In addition, their low specific surface area and long diffusion pathways hinder analyte transport to the sensing interface, thereby reducing response speed and detection sensitivity [8, 9]. Therefore, microscale sensors with low invasiveness, high integration capability, and dimensional compatibility with microenvironments are highly desirable for precision medicine, environmental monitoring, and microfluidic analysis [10, 11, 12].

Nevertheless, scaling down to the micrometer regime introduces critical challenges in signal acquisition and energy supply. The reduced sensing area weakens the output signal, requiring materials with higher intrinsic sensitivity and improved signal to noise ratio [13]. Meanwhile, wired power supplies restrict device layout and adaptability, whereas miniaturized batteries are limited by energy density and cycle life, making long term operation difficult in wearable or implantable systems [14]. Furthermore, real‐world sensing environments often involve the coupling of multiple stress factors, including strain, pressure, temperature, humidity, and chemical substances, that can easily lead to signal crosstalk and reduce detection accuracy. To reduce signal crosstalk caused by multi‐stimulus coupling, multi‐parameter sensing research has, in recent years, gradually developed signal differentiation strategies based on differentiated response mechanisms, independent readout channels, and structural partitioning designs [15, 16]. In sensing systems with temperature‐pressure, temperature‐strain, and temperature‐chemical signal coupling, crosstalk is usually reduced by different functional response units, reference sensing units, or independent output channels [17, 18]. For example, temperature‐pressure dual‐mode sensors can identify temperature and pressure stimuli through thermoelectric output and piezoresistive response, respectively [19]; thermoelectric porous laser‐induced graphene sensors use thermoelectric voltage and resistance changes to respond to temperature gradients and mechanical strain, respectively, to achieve effective differentiation of temperature‐strain signals [20]; in addition, VO*_x_*‐doped laser‐induced graphene multi‐parameter sensors achieve effective differentiation of soil nitrogen loss‐related NO*_x_* signals and temperature signals through encapsulated/unencapsulated structures and self‐heating working modes [21]. These studies show that differentiated response mechanisms, independent signal output channels, and a well‐designed device structure are important approaches to reducing crosstalk in multi‐parameter sensing.

Further targeting practical applications such as flexible wearables and local microenvironment monitoring, sensing systems not only need to effectively distinguish multiple stimulus signals but also possess characteristics such as low power consumption, stable signal output, and convenient integration within a limited size. Therefore, self‐powered sensing technologies that can directly convert external stimuli or environmental energy into electrical signals have attracted widespread attention. Based on different energy conversion mechanisms, self‐powered sensors can be classified into thermoelectric, triboelectric, and piezoelectric types [22, 23, 24, 25]. Compared with triboelectric and piezoelectric sensors, which mainly rely on contact friction or mechanical stress changes, thermoelectric sensors, based on the Seebeck effect, can induce carriers to diffuse directionally from the hot end to the cold end under the action of a temperature gradient, thereby establishing a thermoelectric potential across the material. This thermoelectric potential is directly related to the temperature difference and can therefore serve as an electrical output signal for temperature changes. Thus, under continuous temperature‐difference conditions, thermoelectric sensors can achieve stable, self‐powered signal output without an external power source [26]. However, conventional thermoelectric materials are mainly inorganic semiconductors, whose brittleness, high processing cost, and biocompatibility concerns restrict their use in wearable and implantable systems. In contrast, organic conductive polymers such as polyaniline (PANI), poly(3,4‐ethyldioxothiophene), and polypyrrole exhibit mechanical flexibility, tunable conductivity, processability, and biocompatibility, making them promising for microscale self‐powered sensing despite their relatively lower thermoelectric performance [27, 28, 29].

Among conductive polymers, PANI is particularly attractive because of its reversible doping and dedoping behavior, environmental stability, and intrinsic responsiveness to pH variations and ammonia (NH_3_). However, the relatively low electrical conductivity (*σ)* and Seebeck coefficient (*S*) of pristine PANI result in poor thermoelectric performance, insufficient to enable passive temperature sensing. To enhance the thermoelectric properties of PANI‐based materials, incorporating inorganic fillers such as carbon nanotubes (CNTs), graphene, bismuth telluride particles, and tellurium nanowires (TeNWs) into the PANI matrix has emerged as an effective strategy [30, 31, 32, 33]. Among these, TeNWs are particularly promising due to their high *S* and solution processability, making them compatible with fiber fabrication techniques. Despite progress in related research, existing work remains largely confined to thin film or bulk systems. The challenge persists in transforming high‐performance thermoelectric composites into structurally uniform, integrable micrometer‐scale fiber devices through controllable fabrication—particularly when aiming to combine thermoelectric functionality with chemical sensing capabilities in a single microscale platform.

To address this issue, we report a continuous wet‐spinning technology strategy to fabricate PANI/TeNWs composite microfibers. The optimized fiber achieves a *S* of 59.9 µV K^−1^ and a Power Factor (*PF*) of 9.2 µW m^−1^ K^−2^, enabling passive temperature monitoring with a detection limit of 1 K, where thermal gradients directly generate the sensing signal without external power. Beyond this, the fiber also exhibits excellent chemical sensing capabilities: a Nernstian pH response (59.25 mV pH^−1^) and rapid ammonia detection (0.96 s at 50 ppm). By demonstrating both passive thermoelectric transduction and active chemical sensing in a single microfiber platform, this work provides a material solution that combines energy‐efficient temperature monitoring with high‐performance chemical detection. This sensor, with a diameter of only a few micrometers, achieves high miniaturization while maintaining excellent sensing performance, meeting the signal acquisition needs of confined spaces, complex interfaces, and localized microenvironments. Based on its small size and multifunctional sensing characteristics, this device has potential applications in precision medicine, environmental safety monitoring, and localized condition monitoring in hazardous environments such as fire rescue.

## 2 Results and Discussion

### 2.1 Synthesis and Microstructure

**FIGURE 1:**
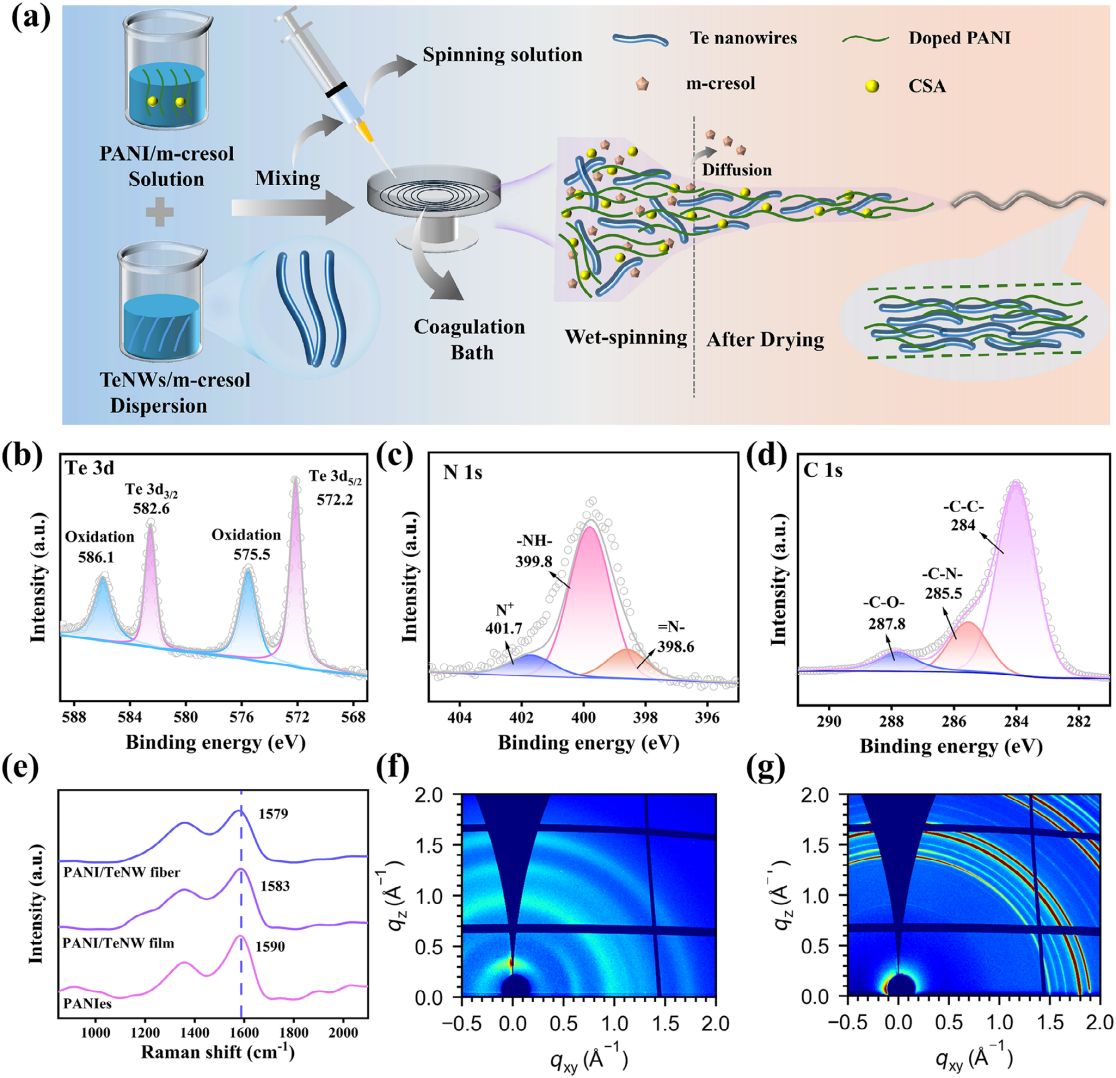
Synthesis and microstructure characterization of PANI/TeNWs composite fiber: (a) Schematic illustration of the fabrication process. (b) Te 3d XPS spectrum of the composite fiber. (c) N 1s and (d) C 1s XPS spectra. (e) Raman spectra of the pristine PANI fiber, PANI/TeNWs film, and PANI/TeNWs fiber. (f, g) GIWAXS patterns of (f) pristine PANI and (g) PANI/TeNWs composite fibers.

The fabrication process of PANI/TeNWs composite fibers is illustrated in Figure 1a. TeNWs were first synthesized via a hydrothermal method and subsequently mixed with camphorsulfonic acid‐doped PANI to form the spinning solution. After thorough dispersion, wet spinning was performed using an ethanol‐coagulation bath. The as‐spun fibers were dried and collected for direct use without any post‐treatment (see Experimental Section for detailed procedures). Digital photograph of the as‐prepared composite fibers are shown in Figure S1a,b. The fiber exhibit excellent flexibility and can be readily knotted, as shown in Figure S1c.

The crystal structure of the synthesized TeNWs was examined by X‐ray diffraction (XRD). As shown in Figure S2, all diffraction peaks are well indexed to the standard pattern of tellurium (JCPDS#36‐1452), confirming the successful synthesis of TeNWs. Fourier transform infrared (FTIR) spectroscopy was employed to characterize the molecular structure of the materials (Figure S3). The emeraldine base polyaniline (PANIeb) exhibits typical characteristic peaks at 828, 1160, 1310, 1499, and 1590 cm^−1^. The peak at 828 cm^−1^ is assigned to the C─H stretching vibration of the benzene ring. The band at 1160 cm^−1^ corresponds to the characteristic absorption of the conducting form of PANI, suggesting electron delocalization along the polymer backbone [34]. The signal at 1310 cm^−1^ is attributed to the C─N stretching vibration. Moreover, the peaks at 1499 and 1590 cm^−1^ are ascribed to C═C stretching vibrations of the benzene ring and quinoid ring, respectively [35].

X‐ray photoelectron spectroscopy (XPS) was performed to gain further insight into the elemental composition and chemical states of the PANI/TeNWs composite fibers. The survey spectrum in Figure S4 confirms the presence of C, N, and Te elements. Figure 1b shows the high‐resolution Te 3d spectrum. In the Te 3d spectrum of PANI/TeNWs, the binding energies at 572.2 and 582.6 eV correspond to the Te 3d_5/2_ and Te 3d_3/2_, respectively. The slight negative shift in Te binding energy compared to pristine Te suggests electron transfer from PANI to TeNWs upon compositing, increasing the electron density around Te atoms and promoting a more reduced state [36]. Additional peaks observed at 575.5 and 586.1 eV are attributed to tellurium oxide (TeO_2_), indicating partial surface oxidation, consistent with the SEM observations. The N 1s spectrum (Figure 1c) can be deconvoluted into three peaks at 398.6, 399.8, and 401.7 eV, corresponding to imine nitrogen (═N‐), amino nitrogen (‐NH‐), and positively charged nitrogen (N^+^) species, respectively, confirming the protonated structure of PANI [37]. The C 1s spectrum (Figure 1d) exhibits characteristic peaks at 284.0, 285.5, and 287.8 eV, assigned to C─C, C─N (from the PANI backbone), and C─O bonds, respectively [38].

Raman spectroscopy was employed to probe the evolution of the conjugated electronic structure within the PANI backbone upon TeNWs incorporation. As shown in Figure 1e, the characteristic band in the 1579–1590 cm^−1^ is attributed to the conjugated backbone C═N/C═C stretching vibrations of the structure in PANI molecules [39]. This vibration is highly sensitive to changes in π‐electron delocalization and conjugation length along the polymer backbone. For pristine PANIes, this characteristic peak is located at ∼1590 cm^−1^, indicating a relatively localized conjugated system in the main chain with limited π‐electron delocalization. Upon introduction of TeNWs, this peak in PANI/TeNWs films red‐shifts to 1583 cm^−1^. In highly oriented PANI/TeNWs composite fibers, the peak further red‐shifts significantly to 1579 cm^−1^. The continuous red shift indicates that the introduction of TeNWs, particularly under the combined effects of fiber‐axis confinement and interfacial interactions, effectively enhances π‐electrons delocalization and extends the conjugation length of the PANI backbone [40].

To further elucidate the molecular packing and orientation features within the composite fibers, grazing‐incidence wide‐angle X‐ray scattering (GIWAXS) measurements were performed. As shown in Figure 1f, the GIWAXS pattern of the pristine PANI exhibits broad, diffuse diffraction rings, indicating isotropic molecular chain packing [41]. In contrast, the PANI/TeNWs composite fibers display significantly enhanced diffraction signals, with the rings gradually evolving into orientation arcs with distinct directional selectivity (Figure 1g). This marked anisotropy primarily originates from the preferential alignment of TeNWs along the fiber axis, given their strong scattering. Simultaneously, the aligned TeNWs provide a structural template that imposes orientational constraints on the PANI molecular chains. Under the synergistic effects of the one‐dimensional fiber confinement and TeNWs templating, the PANI molecular chains undergo conformational stretching and orientation rearrangement along the fiber axis, consistent with the extended conjugation length inferred from the Raman spectroscopy results [42].

**FIGURE 2:**
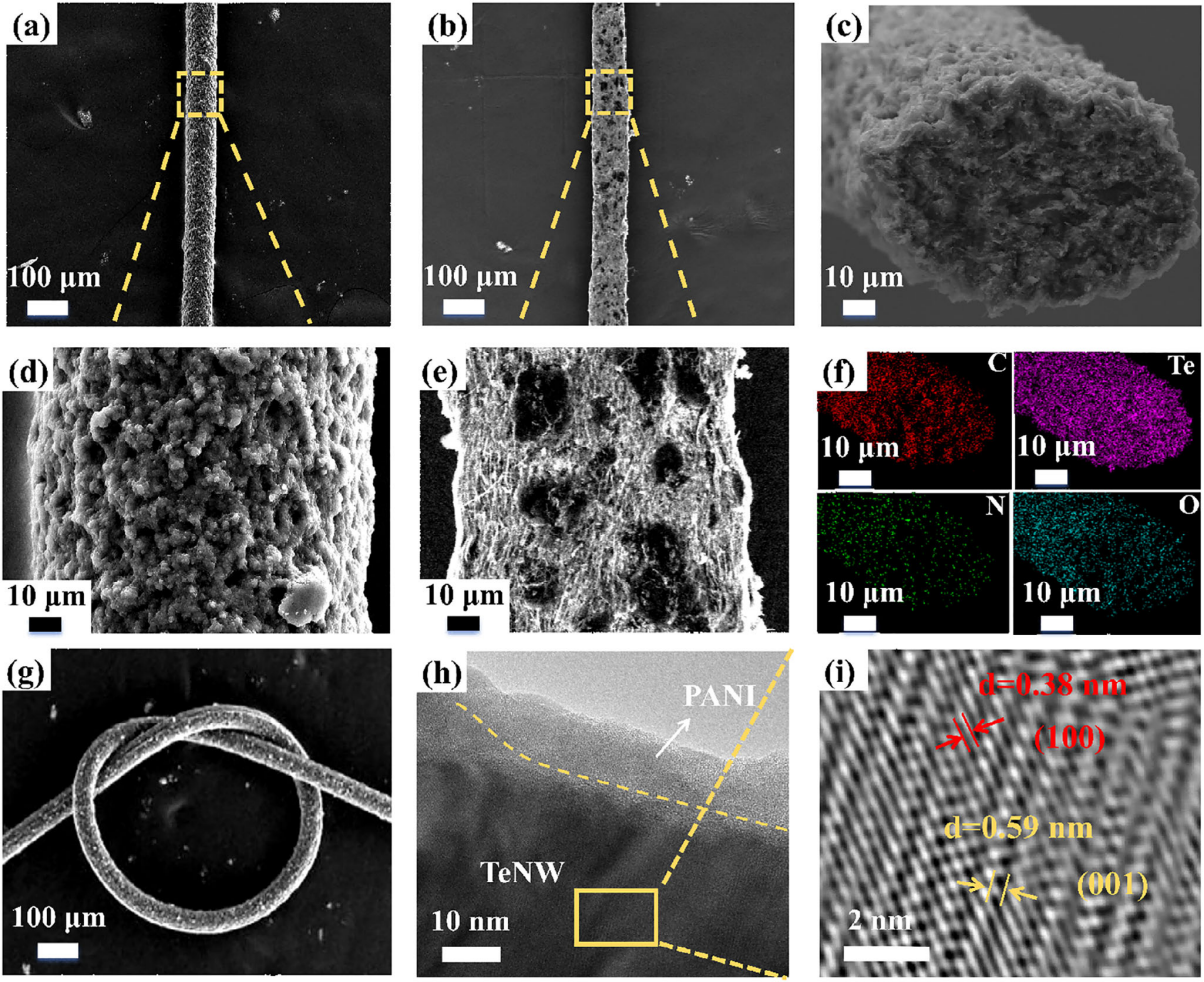
Morphological and microstructural characterization of PANI/TeNWs composite fibers: (a, b) SEM images of (a) pristine PANI and (b) PANI/60 wt.% TeNWs composite fiber. (c) Cross‐sectional SEM image of the composite fiber. (d, e) Enlarged SEM images of (d) pristine PANI and (e) PANI/60 wt.% TeNWs composite fiber surface. (f) EDS spectrum of the PANI/60 wt.% TeNWs composite fiber. (g) Photograph showing a knotted PANI/60 wt.% TeNWs composite fiber. (h) TEM image of the PANI/60 wt.% TeNWs composite fiber. (i) High‐resolution TEM (HRTEM) image revealing lattice fringes of TeNWs within the composite.

The morphology of the composite fibers was examined by scanning electron microscopy (SEM) to gain insight into their microstructural features. Figure 2a,b shows the SEM images of the pristine PANI fiber and PANI/60 wt.% TeNWs composite fiber, respectively. With increasing TeNWs content, the fiber diameter increases from ∼87 to 93 µm, accompanied by a noticeable roughening of the fiber surface. This morphological evolution is attributed to the incorporation of TeNWs, which modifies the rheological properties of the spinning solution [43]. Cross‐sectional SEM imaging coupled with energy‐dispersive X‐ray spectroscopy (EDS) mapping was performed on the composite fiber. The cross‐section exhibits a nearly circular geometry (Figure 2c).

Higher‐magnification SEM images (Figure 2d,e) reveal the presence of surface pores on both pristine PANI and PANI/TeNWs fibers. These pores originate from a double diffusion process between the solvent (m‐cresol) and the ethanol coagulation bath during spinning: ethanol diffuses into the fiber interior and subsequently volatilizes during drying, leaving behind a porous structure [44]. Such porosity is beneficial for enhancing the accessibility of analytes, potentially improving the sensitivity of subsequent gas and pH sensing. Furthermore, Te, C, N, O, and S elements on the cross‐sectional area are uniformly distributed (Figure 2f and Figure S5), confirming the successful and homogeneous incorporation of TeNWs within the PANI matrix. Despite the high TeNWs loading of 60 wt.%, the composite fibers exhibited excellent flexibility and could be easily knotted, as illustrated in Figure 2g. Transmission electron microscopy (TEM) was employed to further investigate the microstructure of the PANI/TeNWs composite fiber. Low‐magnification TEM images (Figure 2h) reveal TeNWs embedded and enveloped within the PANI matrix. High‐resolution TEM (HRTEM) imaging (Figure 2i) reveals distinct lattice fringes with interplanar spacings of 0.38 and 0.59 nm, corresponding to the (100) and (001) crystal planes of hexagonal Te, respectively [45]. These observations confirm the high crystallinity of the TeNWs and their intimate encapsulation by PANI, indicating effective interfacial contact between the two components—a key factor for achieving synergistic thermoelectric and sensing performance.

### 2.2 Thermoelectric Performance

**FIGURE 3:**
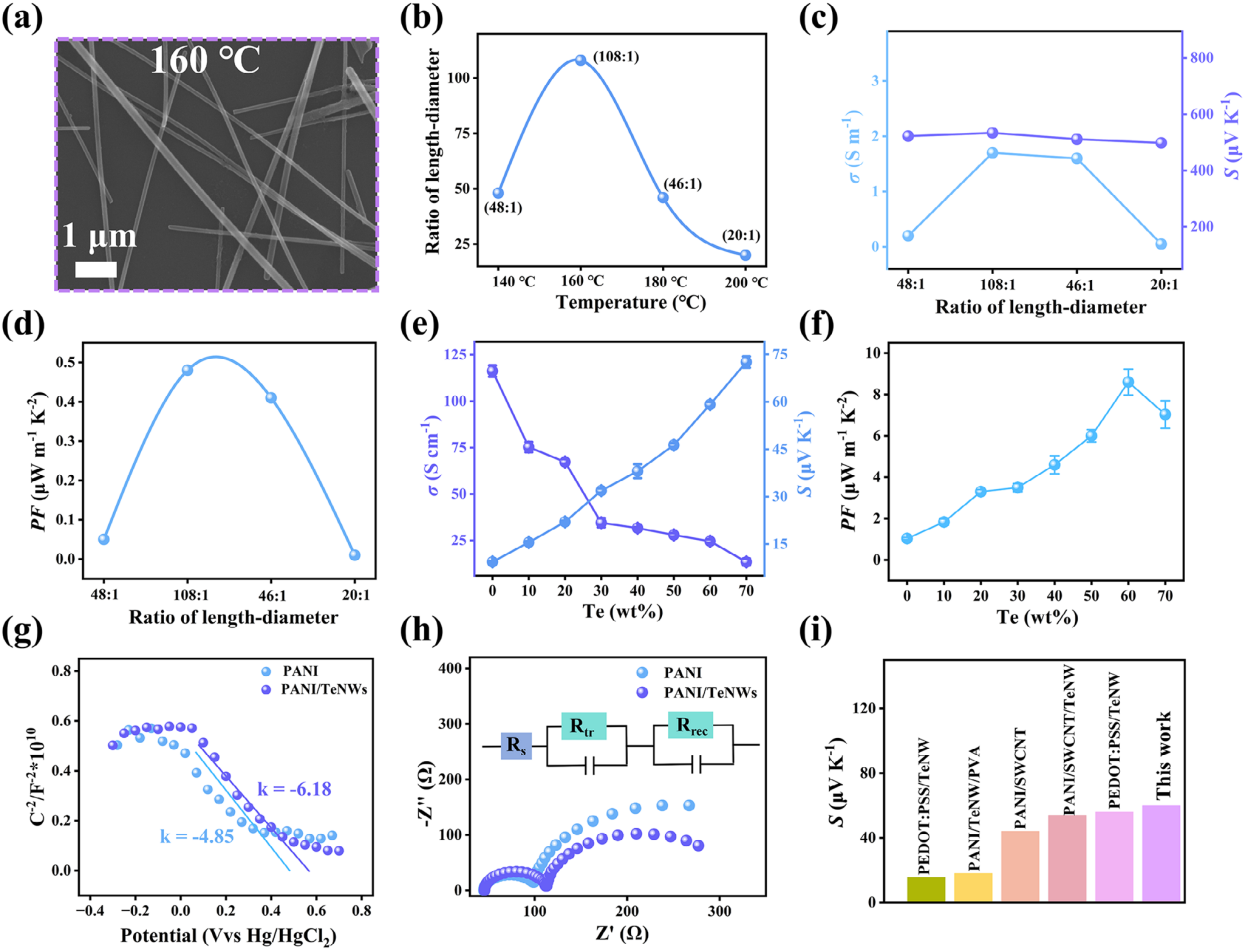
Thermoelectric properties of PANI/TeNWs composite fibers: (a) SEM images showing the morphology of TeNWs synthesized at 160 ℃. (b) Aspect ratio of TeNWs plotted against synthesis temperature. (c, d) Electrical conductivity, *σ*, Seebeck coefficient, *S* (c), and corresponding power factor, *PF* (d) of TeNWs with different aspect ratios. (e, f) Electrical conductivity, *σ*, Seebeck coefficient, *S* (e), and corresponding power factor, *PF* (f) of PANI/TeNWs composite fibers, showing a maximum at 60 wt.% TeNWs. (g, h) Mott‐Schottky plots for comparing the carrier concentration (g) and Nyquist plots (h) of pristine PANI and PANI/60 wt.% TeNWs composite fibers. (i) Benchmarking of the Seebeck coefficient achieved in this work against values reported for other PANI‐based composites in the literature.

The aspect ratio of TeNWs is a key structural parameter influencing their thermoelectric properties, as it significantly influences carrier transport behavior [46]. SEM images of TeNWs synthesized at different temperatures are shown in Figure 3a and Figure S6a–c, with corresponding length and diameter distributions provided in Figure S7a–h. As the temperature increased, the aspect ratios were determined to be 48 : 1, 108 : 1, 46 : 1, and 20 : 1, respectively, reaching a maximum at 160 ℃ (Figure 3b). TeNWs with different aspect ratios were vacuum‐filtered into films, and their thermoelectric properties were evaluated (Figure 3c,d). The results demonstrate that the sample synthesized at 160 ℃ exhibits the optimal thermoelectric performance. Accordingly, TeNWs synthesized at 160 ℃ were selected for subsequent composite formation with PANI.

To elucidate the influence of TeNWs loading on the thermoelectric properties of PANI/TeNWs composites, the*σ*, *S*, and *PF* as a function of TeNWs content were systematically examined (Figure 3e,f). With increasing TeNWs content, the *S* significantly improved from 10.1 to 75.8 µV K^−^^1^, representing a sevenfold improvement. According to Equation (1) [47]:

(1) $$S=\frac{8\pi^{2}\kappa_{B}^{2}}{3eh^{2}}m^{*}T{\left(\frac{\pi }{3p}\right)}^{\frac{2}{3}}$$

where *e* is the elementary charge, *h* Planck constant, *κ*_B_ Boltzmann of constant, *p* the carrier concentration, m^*^ the effective mass of the charge carriers, and *T* the absolute temperature. This relation indicates that *S* is inversely proportional to *p*. To probe the change in carrier concentration, Mott‐Schottky analysis was performed using impedance spectroscopy. The Mott–Schottky relationship is given by Equation (2) [48]:

(2) $$\frac{1}{C_{\mathrm{sc}}^{2}}=\frac{2}{\mathrm{eN}\varepsilon \varepsilon_{0}A^{2}}\left(V_{m}-V_{\mathrm{fb}}-\frac{k_{B}T}{e}\right)$$

where *C_SC_* denotes the space‐charge capacitance, *V_m_* the applied voltage, *V_fb_* the flat‐band potential, *N* the carrier concentration, *ε* the relative permittivity, *ε*_0_ the vacuum permittivity, *A* the sample‐liquid contact area, and *k_B_T/e* ∼25 mV at room temperature (negligible). The Mott–Schottky plots obtained for pristine PANI and PANI/TeNWs composite fibers are shown in Figure 3g. Linear fitting of the linear region yields the slope *k*, which is related to *N* by Equation (3) [49]:

(3) $$k=\frac{-2}{\mathrm{eN}\varepsilon \varepsilon_{0}A^{2}}$$

Since all samples were measured using the same electrode area and identical testing configuration, *A* ^2^ can be regarded as a constant for relative comparison. Accordingly, the variation in the apparent carrier concentration can be qualitatively evaluated from the absolute value of the Mott–Schottky slope, leading to Equation (4):

(4) $$N\propto \frac{-2}{\mathrm{ek}\varepsilon \varepsilon_{0}}$$

Thus, a steeper slope corresponds to a lower carrier concentration. As can be seen from the Figure 3g, the absolute value of the slope increases from 4.85 for pristine PANI to 6.18 for the PANI/TeNWs composite fiber, confirming that the incorporation of TeNWs reduces the carrier concentration, thereby enhancing the *S*.

Despite the increase in *S*, the electrical conductivity of the composites gradually decreased with rising TeNW content (Figure 3e). The intrinsic *σ* of the TeNWs is only 0.017 S cm^−1^. Their introduction disrupts the continuous conductive network of PANI, reducing the density of effective charge transport pathways [43]. Furthermore, the increased organic‐inorganic interfaces alter charge carrier mobility. According to Equation (5):

(5) σ=eμN

where *µ* signifies the carrier mobility. To assess the mobility change, electrochemical impedance spectroscopy (EIS) was performed (Figure 3h). The Nyquist plots exhibit two semicircles, fitted with an equivalent circuit comprising series resistance (*R*_s_), charge transfer resistance (*R*_tr_), and recombination resistance (*R*_rec_). The first semicircle represents the *R*_tr_, and the second semicircle represents the *R*_rec_ [50]. After TeNWs incorporation, *R*_tr_ increases while *R*_rec_ decreases, indicating reduced carrier mobility. Therefore, the decrease in conductivity arises from the synergistic reduction of both carrier concentration and mobility upon TeNW incorporation. At a TeNW loading of 60 wt.%, the composite fiber achieved a maximum *PF* of 9.2 µW m^−1^ K^−2^. The spinnability deteriorated when the TeNW content exceeded 70 wt.%, leading to discontinuous fiber formation (Figure S8). Therefore, the PANI/60 wt.% TeNWs composite fiber was selected for further structural characterization and application studies. Furthermore, fibers spun using coarse needles exhibit a relatively higher *PF* compared to fine fibers (Figure S9a–c). This may be attributed to the reduced removal of the Camphorsulfonic acid (CSA) phase in fibers due to spatial constraints [51]. However, coarse fiber preparation with 22G needles may exhibit diminished thermoelectric performance due to increased structural defects arising from lower shear rates and non‐uniform solidification. The reproducibility of the thermoelectric properties of the PANI/TeNWs composite fibers was further evaluated by measuring *σ* and *S* of individual fiber samples and independently prepared batches (Figure S10). As shown in Figure S10a, the five individual fiber samples yield average *σ* = 22.3 ± 3.0 S cm^−1^ and *S* = 57.9 ± 2.7 µV K^−1^, corresponding to relative standard deviations (RSD) of 13.5% and 4.7%, respectively, indicating good sample‐to‐sample uniformity. In addition, the batch‐to‐batch comparison among three independently prepared batches shows consistent thermoelectric properties, with average *σ* and *S* values of 21.5 ± 2.1 S cm^−1^ and 58.9 ± 2.7 µV K^−1^, respectively (Figure S10b). The small error bars confirm the good fabrication reproducibility of the wet‐spun composite fibers. Figure 3i compares the *S* of recently reported PANI‐based composites; the PANI/TeNWs fiber significantly outperforms the other composites, underscoring the effectiveness of our organic‐inorganic composite strategy [51, 52, 53, 54, 55].

### 2.3 Thermoelectric Device Demonstration and Passive Temperature Sensing

**FIGURE 4:**
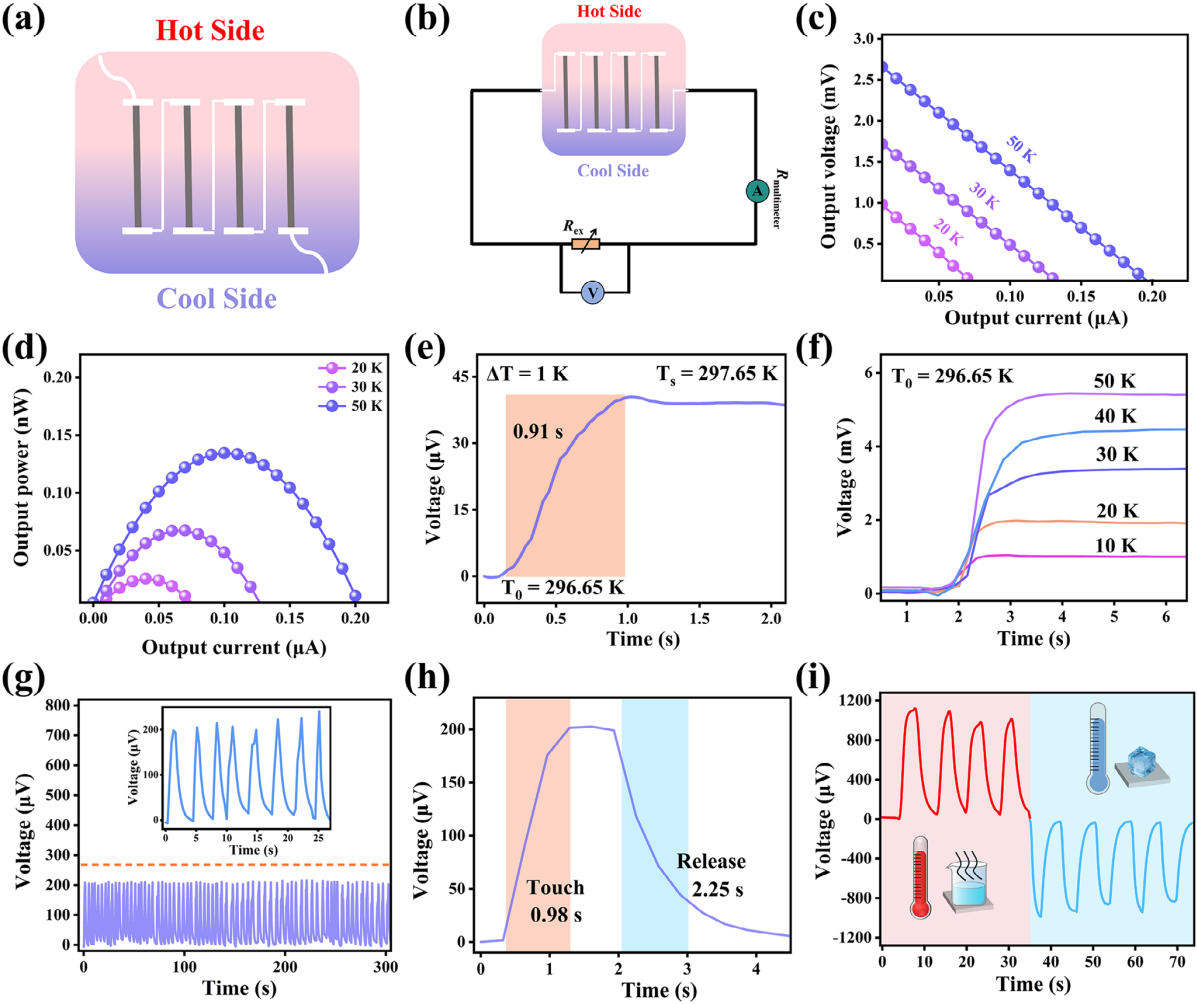
Device configuration and demonstration of passive temperature sensor: (a) Schematic of the sensor device based on a single‐legged PANI/TeNWs composite fiber. (b) Circuit schematic used for measuring the thermoelectric output. (c, d) Open‐circuit voltage (c) and output power (d) measured at temperature differences (Δ*T*) of 20 to 50 K. (e) Real‐time Voltage response showing a clear signal at a Δ*T* of 1 K, demonstrating the low detection limit. (f) Voltage response as Δ*T* incrementally increased from 10 to 50 K. (g) Stability test showing consistent voltage output during repeated finger contact over 300 s. (h) Dynamic response of the sensor, showing a rapid response time of 0.98 s and recovery time of 2.25 s upon finger touch. (i) Voltage response to opposite temperature gradients, showing a positive signal for the hot stimulus (beaker of hot water) and a negative signal for the cold stimulus (ice).

For thermoelectric temperature sensors, the output voltage amplitude typically satisfies the condition:

(6) $$|V_{TE}|=\left|S\right|\;\Delta T$$

where *V_TE_* is the thermoelectric output voltage, *S* is the Seebeck coefficient, and Δ*T* is the temperature difference between the two ends of the material. Therefore, under the same temperature difference, a larger absolute value of the Seebeck coefficient can produce a higher thermoelectric output voltage amplitude, making the electrical signal induced by a small temperature difference easier to distinguish. Based on this characteristic, PANI/TeNWs composite fibers were used to fabricate temperature sensors to evaluate their thermoelectric output capability under temperature gradients (Figure S11). Individual fibers were electrically connected using silver paste, with both ends bonded to silver wires using the same conductive material, as illustrated in Figure 4a. For thermoelectric characterization, one end of the sensor was placed on a temperature‐controlled hot plate while the opposite end remained exposed to ambient air, establishing a steady‐state temperature gradient along the fiber length (Figure 4b). Figure 4c,d shows the current dependence of output voltage/power under temperature differences (Δ*T*) of 20–50 K. As Δ*T* increased from 20 to 50 K, the output voltage increased from 0.98 to 2.66 mV. To determine the output power, external resistors of varying resistances were connected in series with the device. The output power *P* was calculated according to Equation (7) [56]:

(7) $$P=\frac{E^{2}}{\frac{{\left(R_{\mathrm{ex}}-R_{\mathrm{in}}\right)}^{2}}{R_{\mathrm{ex}}}+4R_{\mathrm{in}}}$$

where *E* is the open‐circuit voltage of the device, *R*_ex_ is the external resistance, and *R*_in_ is the total internal resistance. For a fixed Δ*T*, *P* depends on *R*_ex_, reaching a maximum when *R*_ex_ equals *R*_in_. As Δ*T* increased from 20 to 50 K, the maximum *P* rose from 0.03 to 0.13 nW.

The temperature response sensitivity is shown in Figure 4e, demonstrating a minimum detectable temperature difference of 1 K. Figure 4f displays the output voltage as a function of Δ*T*, confirming a monotonic, nearly linear increase with increasing Δ*T*. The cyclic stability of the sensor was evaluated over 300 s (Figure 4g); the stable and reproducible output voltage upon repeated thermal cycling confirms its reliability for dynamic temperature monitoring. Although the sensor overall response was stable and repeatable, slight baseline drift and peak fluctuations still occurred during cyclic testing. This was primarily attributed to the dynamic heat transfer process and differences in manual contact conditions. During finger contact and removal, the establishment and recovery of the local temperature field are not entirely instantaneous, and residual heat accumulation during continuous testing may cause slight baseline drift. Simultaneously, differences in contact area, pressure, location, and time alter the effective temperature gradient, thus causing peak fluctuations. The practical applicability of the sensor was further demonstrated through finger touch tests (Figure 4h). Upon finger contact, the sensor generated an output voltage of ∼200 µV, with rapid response and recovery times of 0.98 and 2.25 s, respectively. Additionally, the sensor responded distinctly to both hot and cold stimuli (Figure 4i). When a beaker containing hot water is placed at one end of the sensor, a positive voltage signal is generated; conversely, when ice is placed, the sensor produces a negative voltage signal. These results confirm the capability of the sensor for passive, real‐time temperature monitoring across a wide range of thermal conditions.

### 2.4 pH Sensing Performance of Probe Sensors

Monitoring local microenvironment pH requires sensors capable of contacting restricted, narrow, or irregularly shaped areas and accurately capturing local pH signals. Compared to traditional bulk or planar film‐type pH sensors, micro‐probe sensors offer superior local contact and adaptability to the microenvironment, thereby reducing the signal averaging effect caused by large‐area detection. Therefore, fibrous sensing materials, which combine small size, structural flexibility, and ease of local contact, are better suited as sensing platforms for localized pH detection and microenvironment analysis [57].

Based on the reversible doping/dedoping characteristics of PANI, the PANI/TeNWs composite fibers hold significant promise for pH sensing [58]. The doping mechanism of PANI involves the incorporation of protons (H^+^) and counterions (e.g., Cl^−^, sulfate, phosphate) from dissociated protonic acids into the polymer backbone. These ions interact with nitrogen atoms in amine and imine groups, forming polarons and bipolarons that substantially enhance electrical conductivity through π‐electron delocalization. Under alkaline conditions, deprotonation triggers a de‐doping process, restoring the polymer to its initial state [59]. The PANI/TeNWs probe‐type pH sensors based on this mechanism operate by measuring changes in Open Circuit Potential (OCP), which exhibits a linear relationship with H^+^ concentration as described by the Nernst equation:

(8) $$E_{1}=E_{0}+\frac{\mathrm{RT}}{F}\ln{\left[H\right]}^{+}$$

where *E*_0_ is the standard electrode potential, *R* is the molar gas constant, and *F* is faraday constant.

**FIGURE 5:**
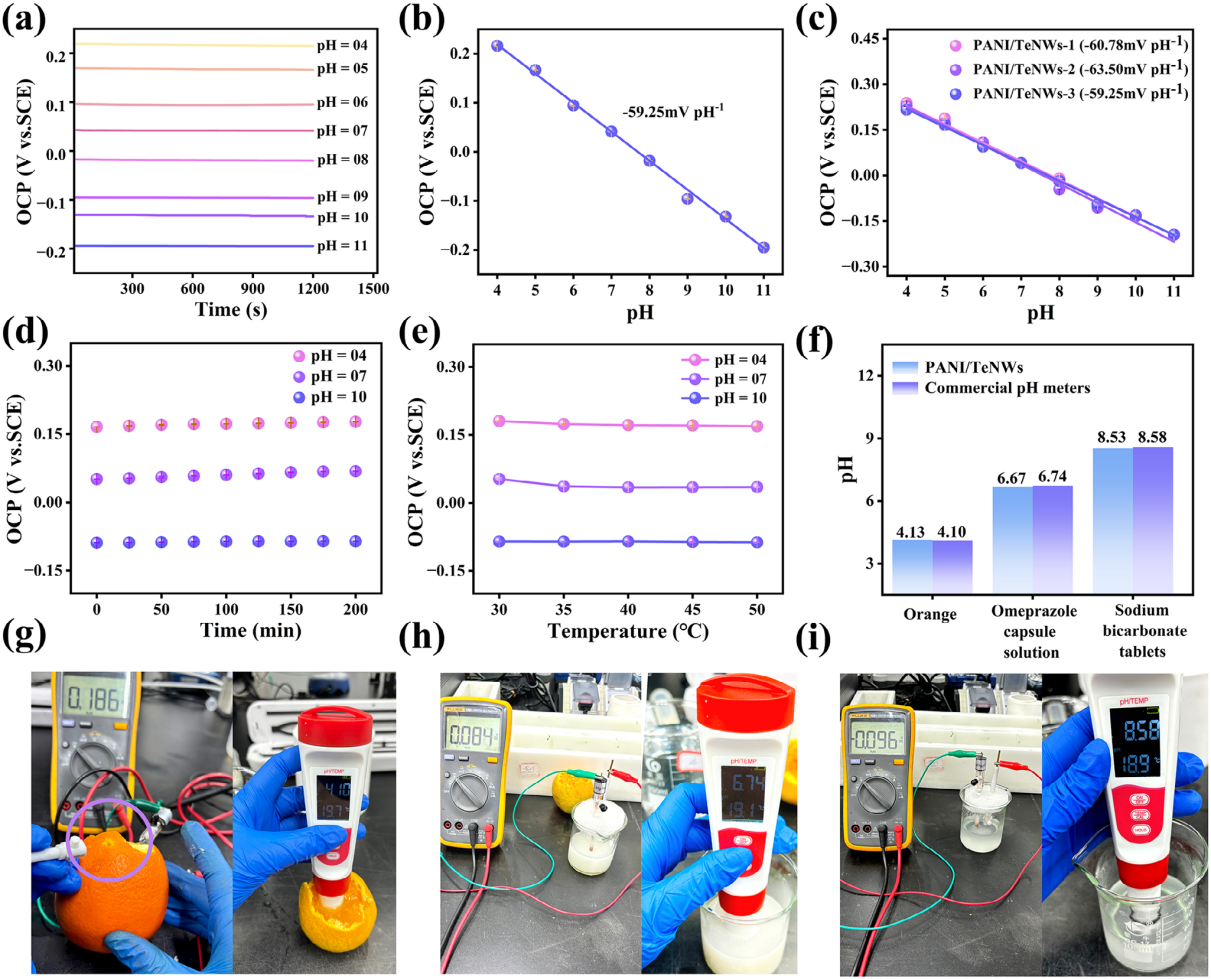
Evaluation of the PANI/TeNWs fiber sensor for pH sensing: (a) Real‐time open‐circuit potential, OCP response upon stepwise changes in pH from 4 to 11. (b) Calibration curve showing a linear relationship between OCP and pH, with a sensitivity of 59.25 mV pH^−1^, closely matching the theoretical Nernstian value (59.16 mV pH^−1^). (c) Reproducibility of three independent sensors, demonstrating consistent sensitivity across the pH range. (d) Potential drift of the sensor over 200 min at pH 4, 7, and 10, indicating good long‐term stability. (e) Relationship between measured potential and temperature at different pH values (4, 7, and 10). (f) Comparison of pH values obtained from the PANI/TeNWs sensor and a commercial pH meter for various real‐world samples, demonstrating excellent agreement. (g–i) Digital photographs and corresponding pH measurements for (g) orange juice, (h) omeprazole enteric‐coated capsule solution, and (i) sodium bicarbonate tablet solution, validating practical applicability.

The pH sensing performance of the PANI/TeNWs composite fiber was evaluated by monitoring OCP across a buffer solution range of pH 4 to 11 (Figure 5a). The sensor exhibited a linear response with a sensitivity of 59.25 mV pH^−1^ (*R^2^* = 0.99), closely matching the theoretical Nernstian value of 59.16 mV pH^−1^ at room temperature (Figure 5b). This near‐ideal response confirms the effective protonation/deprotonation behavior of the PANI matrix within the composite. Repeatability was assessed by measuring three independently fabricated sensors over the same pH range (Figure 5c). The sensitivities ranged from 59.25 to 63.50 mV pH^−1^, with an average sensitivity of 61.17 mV pH^−1^ and a relative standard deviation (RSD, *n* = 3) of 3.5%. Despite slight variations in absolute potential, the consistent sensitivity demonstrates the reliable H^+^ detection capability of the PANI/TeNWs sensor.

The long‐term stability and potential drift were examined by monitoring OCP in buffer solutions at pH 4, 7, and 10 (corresponding to weakly acidic, neutral, and weakly alkaline conditions, respectively) over 200 min, with data recorded every 25 min (Figure 5d). After 200 min, the potential drifts were 11.1 (pH 4), 17.2 (pH 7), and 3.0 mV (pH 10). These drifts are attributed to gradual ion migration and hydration effects at the sensor‐electrolyte interface. Temperature dependence of the sensor response was also investigated (Figure 5e). Within the temperature range of 30–50 ℃, the OCP change rates were 0.57, 0.89, and 0.09 mV at pH 4, 7, and 10, respectively. Elevated temperatures accelerate the dissociation of weak acid‐base pairs, altering H^+^ concentration and thus shifting the measured potential. These results highlight the necessity of temperature compensation for accurate pH measurements in practical applications, particularly under varying thermal conditions.

Interference studies were conducted by introducing potential interfering species into the buffer solutions across a concentration range of 0–15 mm (Figure S12). For common cations, NH_4_^+^, Ca^2+^, Na^+^, and K^+^, their chloride salts were used. Their OCP values exhibited minimal variation with increasing concentrations of these cations, indicating good selectivity of the PANI/TeNWs sensor toward H^+^, over other physiologically relevant ions. In a separate experiment, MgO was introduced to assess its response under alkaline perturbations. Upon addition, MgO reacts with water to form Mg(OH)_2_, which dissociates and increases the local OH^−^ concentration, thereby altering the solution pH. Consequently, a significant potential shift was observed at 15 mm MgO, attributable to the pH change induced by Mg(OH)_2_ formation rather than direct ionic interference. This behavior further confirms that the sensor responds primarily to H^+^ activity, and any substantial potential drift in the presence of MgO arises from its effect on solution pH rather than from a lack of selectivity.

When paired with a saturated calomel electrode (SCE) reference electrode, the measured pH values were consistent with those from commercial pH meters (Figure 5f), confirming its accuracy and reliability. To validate practical applicability, the PANI/TeNWs sensor was tested on real samples, including orange juice, omeprazole enteric‐coated capsules, and sodium bicarbonate tablets, as shown in Figure 5g–i. Compared with previously reported pH sensors, the PANI/TeNWs composite fiber exhibits pH response sensitivity close to the Nernst theoretical value across a wide pH detection range, while demonstrating low measurement RSD and, most notably, excellent localized micro‐area detection capabilities, further highlighting its application potential in practical pH monitoring applications and microenvironment analysis (Table S1). Based on the key parameters summarized in Table S1, a radar chart was constructed to compare the overall performance of different pH sensors (Figure S13). Among the compared sensors, the sensor developed in this work shows the largest normalized area in the radar chart, suggesting a more balanced performance across the evaluated metrics. Although some reported sensors exhibit advantages in a single metric, the present sensor achieves a favorable balance among near‐Nernstian response, pH detection range, satisfactory repeatability, and micro‐area detection capability, highlighting its balanced overall pH sensing performance.

### 2.5 Ammonia Gas Sensing Performance

**FIGURE 6:**
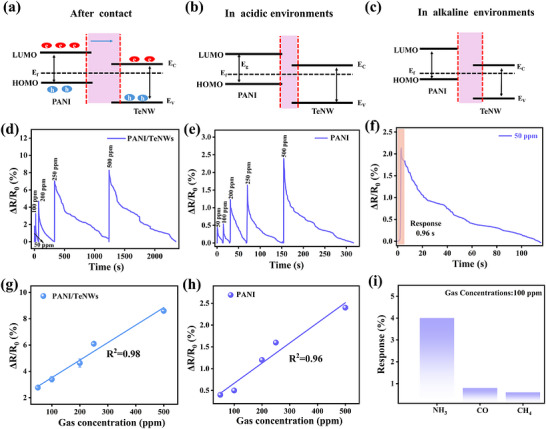
Evaluation of the PANI/TeNWs fiber sensor for ammonia sensing: (a) Energy band diagram showing the heterojunction between PANI and TeNWs and the formation of a depletion region. (b, c) Schematic diagrams depicting the reversible modulation of the depletion layer width in (b) acidic (HCl) and (c) alkaline (NH_3_) environments. (d, e) Real‐time resistance response of (d) the PANI/TeNWs composite sensor and (e) the pristine PANI fiber sensor upon exposure to varying concentrations of NH_3_ (50–500 ppm). (f) Enlarged view of the response transient to 50 ppm NH_3_, revealing a rapid response time of 0.96 s. (g, h) Corresponding calibration curves showing the linear relationship between response and NH_3_ concentration for (g) the PANI/TeNWs sensor and (h) the pristine PANI sensor. (i) Selectivity test comparing its response to 100 ppm NH_3_ against 100 ppm CO and CH_4_, demonstrating excellent selectivity for ammonia.

Using the unique chemical properties of PANI, the fiber also functions as an efficient gas‐sensing material. Both PANI and TeNW are p‐type semiconductors; their energy band alignment prior to contact is illustrated in Figure S14. Upon interfacial contact, holes diffuse from the side with the higher Fermi level to that with the lower Fermi level, forming a stable depletion region at the heterojunction (Figure 6a). When exposed to different gaseous environments, the depletion layer width in the PANI/TeNWs composite fiber changes significantly. In an acidic environment (e.g., HCl vapor), hydrogen ions (H^+^) interact with the composite, reducing the depletion layer thickness (Figure 6b). Conversely, in an alkaline environment (e.g., NH_3_ gas), hydroxide ions (OH^−^) increase the depletion layer width (Figure 6c).

Given the fine diameter of individual fibers, ten fibers were stacked to fabricate a sensing device as shown in Figure S15. Gas sensing tests were performed at room temperature over an NH_3_ concentration range of 50 to 500 ppm, with the experimental setup depicted in Figure S16. Figure 6d shows the dynamic response curves across this concentration range, where the response amplitude increases monotonically with rising NH_3_ concentration. Compared to the pristine PANI fiber sensor, the TeNWs‐doped sensor exhibits significantly enhanced response values under identical test conditions (Figure 6e), indicating that TeNWs incorporation improves the sensing performance. Remarkably, the sensor achieves a response time of ∼0.96 s upon exposure to 50 ppm NH_3_ (Figure 6f). Figure 6g,h compares the response accuracy of the PANI/TeNWs and pristine PANI sensor across 50–500 ppm, revealing the superior performance of the composite sensor. At a fixed concentration of 100 ppm, the sensor exhibits distinct response characteristics toward different gas types; its response to NH_3_ is substantially higher than to CO and CH_4_ (Figure 6i), indicating selectivity toward NH_3_. Compared with reported gas‐sensing materials, the PANI/TeNWs composite fiber sensor developed in this work enables NH_3_ detection over a concentration range of 50–500 ppm at room temperature, while exhibiting a short response time of 0.96 s and good linearity. In addition, the sensor maintains a stable NH_3_ response after 30 days of storage, indicating its good sensing stability during storage (Table S2).

To further evaluate the environmental adaptability and mechanical stability of the PANI/TeNWs composite fiber sensor under actual service conditions, the effects of temperature changes and bending deformation on its NH_3_ sensing performance were further investigated. As shown in Figure S17a, the sensor maintains a clear response to 200 ppm NH_3_ at 15 ℃, 25 ℃, 45 ℃, and 65 ℃, indicating stable NH_3_ sensing capabilities under different thermal environments. The temperature‐dependent recovery behavior can be attributed to the accelerated desorption and diffusion of NH_3_ molecules at elevated temperatures. In this process, reduced adsorption of NH_3_ at the fiber interface reduces its deprotonation/dedoping effect on protonated PANI, allowing partially dedoped PANI chains to recover their protonated state gradually and thereby restoring the device resistance to its initial value. In addition, the sensor exhibits a repeatable response after bending (Figure S17b), suggesting that the PANI/TeNWs composite fiber maintains continuous conductive pathways. These results further demonstrate its mechanical reliability and its potential for flexible NH_3_ sensing applications.

To further verify the response capability of the sensor under coexisting temperature variation and chemical stimuli, its output signals were measured under different temperature gradients during exposure to 200 ppm NH_3_ (Figure S18a–c). The results show that the sensor can simultaneously generate thermoelectric voltage signals corresponding to the temperature gradient and relative resistance change signals induced by NH_3_ exposure. This indicates that the sensor can respond to temperature and chemical stimuli through two independent output signals, demonstrating its potential for simultaneous temperature/chemical monitoring applications.

The enhanced gas sensing performance of the PANI/TeNWs composite fiber gas sensor can be attributed to synergistic contributions from both components. On one hand, PANI in its emeraldine salt form acts as a p‐type semiconductor. NH_3_ molecules, functioning as electron donors, can serve as de‐dopants for PANI. Upon adsorption onto the PANI surface, NH_3_ reacts with imine groups to form ammonium ions (NH_4_^+^). This process localizes polarons within PANI backbone, increasing the sensor resistance and converting PANI from the conductive emeraldine salt to the less conductive emeraldine base form. The reaction is reversible: NH_4_^+^ decomposes into NH_3_ and H^+^, allowing the sensor resistance to recover upon removal of the analyte. On the other hand, the tellurium oxide layer formed on the TeNWs surface (as evidenced by XPS in Figure 1b) also contributes to ammonia sensing. NH_3_ molecules, as an electron donor, can reduce the surface tellurium oxide back to elemental tellurium, thereby lowering the majority carrier concentration in TeNWs and reducing the overall conductivity of the composite fiber [60]. This dual sensing mechanism—involving both PANI dedoping and TeNWs surface reduction, accounts for the superior sensitivity and rapid response of the PANI/TeNWs composite toward NH_3_.

### 2.6 Environmental and Mechanical Stability

**FIGURE 7:**
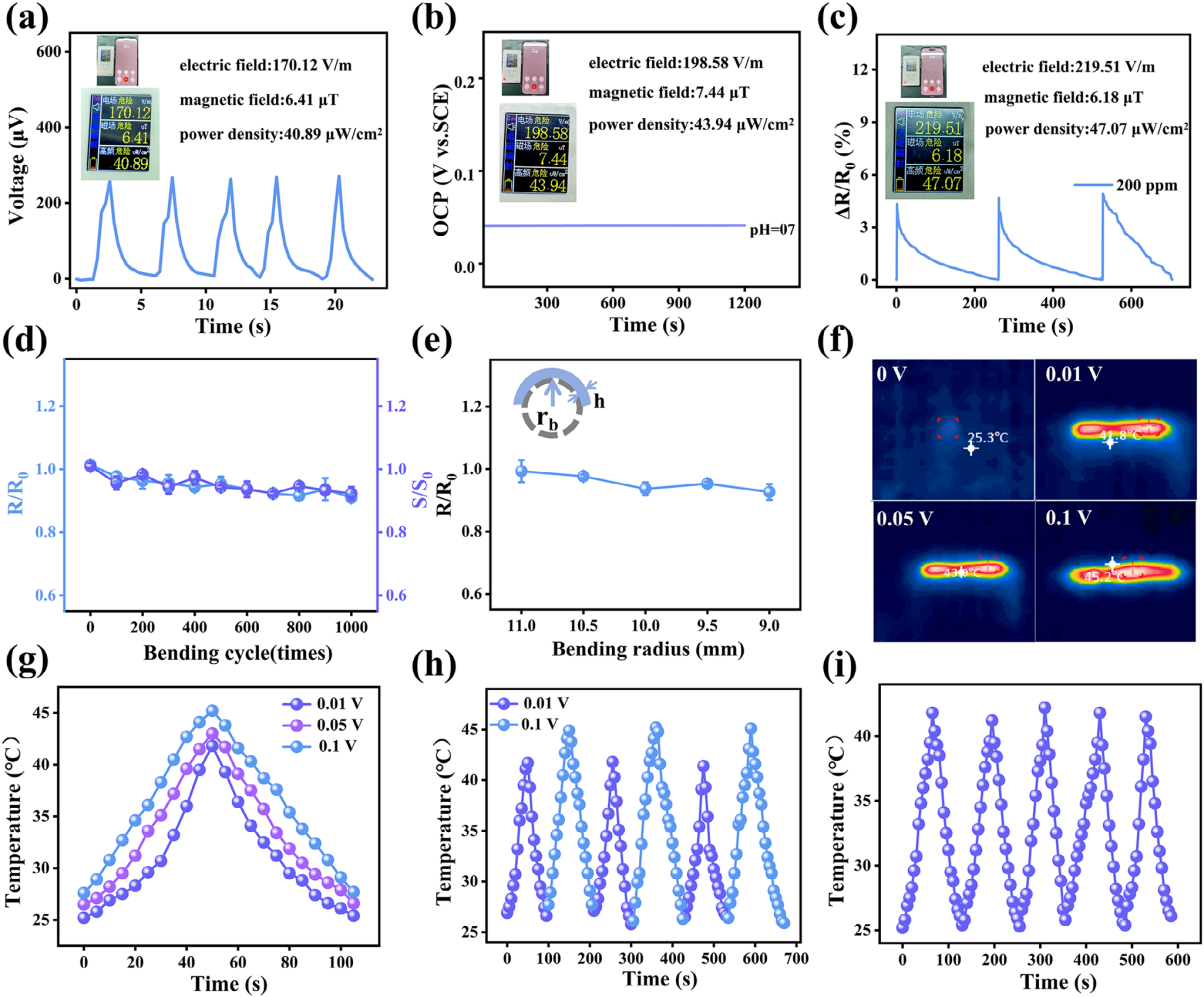
Stability evaluation and Joule Heating Performance of PANI/TeNWs fibers: (a‐c) Validation of sensing reliability under electromagnetic interference: comparison of (a) temperature, (b) pH, and (c) gas sensing signals measured in an electromagnetically shielded environment and near an active mobile phone, demonstrating excellent immunity to electromagnetic fields. (d) Mechanical durability: normalized resistivity (*R*/*R*_0_) and Seebeck coefficient (*S*/*S*_0_) of the composite fiber after repeated bending (100 to 1000 cycles) at a 90° angle, showing less than 10% degradation. (e) Flexibility test: normalized resistivity (*R*/*R*_0_) of the fiber at various bending radius (*r_b_*). (f) Infrared thermal images capturing the surface temperature distribution of the fiber device under different applied voltages. (g) Real‐time temperature increase and saturation profiles at applied voltages. (h) Reversible temperature modulation upon cyclic switching of the applied voltage between 0.01 V and 0.1 V. (i) Cyclic stability of Joule heating performance over five on/off cycles at 0.01 V, confirming excellent reproducibility.

Given the ubiquitous presence of electromagnetic wave environment in everyday environments, the sensing output of the PANI/TeNWs sensor under different electromagnetic field conditions was evaluated to verify its operational stability. Figure 7a–c presents the results obtained in the presence of electromagnetic interference (i.e., near an active mobile phone during a call). The output signals measured under these conditions were essentially identical to those measured in the field‐free environment, demonstrating that electromagnetic waves exert a negligible effect on the sensing performance. This insensitivity is primarily attributed to the low‐frequency characteristics of common electromagnetic sources, which do not induce significant thermal effects or temperature fluctuations that could otherwise perturb the thermoelectric voltage.

To evaluate the durability of the composite fibers under practical wearing conditions, the influence of bending cycles and bending radius on their electrical performance was investigated. Figure 7d shows the normalized resistivity change (*R*/*R*_0_) of fibers bent manually at a 90°, where *R* represents the resistance in the bent state and *R*_0_ denotes the resistance in the straight state. The normalized resistivity change remained below 10% throughout bending. After 100 to 1000 bending cycles, the *S* retained more than 90% of its initial value, indicating excellent flexibility and bending stability of the fibers. The dependence on bending radius was further investigated by placing PANI/TeNWs onto glass tubes of different diameters (Figure 7e). The composite fiber exhibited a resistivity change of less than 10% at a bending radius of 9 mm, confirming its good flexibility and stability. The environmental stability of the PANI/TeNWs composite fiber was further evaluated by monitoring its normalized resistance during exposure to ambient air (Figure S19). The *R*/*R*_0_ value remains close to 1.0 with only a slight increase over 9 days, indicating that the conductive network of the composite fiber is largely preserved throughout the exposure period. This stable resistance behavior suggests good environmental stability and supports its reliability for practical sensing applications.

To further evaluate the long‐term stability of the sensor in air, its sensing performance was tested after different storage periods (Figure S20). After 30 days, the sensor still maintained a stable thermoelectric voltage response, exhibited a stable OCP output in a pH 4 buffer solution, and showed a distinct resistance response to 200 ppm NH_3_. Specifically, the average OCP value in the pH 4 buffer solution changed from 0.22 V initially to 0.23 V after 30 days, corresponding to only a 10 mV potential shift. For 200 ppm NH_3_, the response values on days 1, 9, and 30 were 4.39%, 4.43%, and 4.54%, respectively. Compared with day 1, the responses on days 9 and 30 increased only slightly by approximately 0.91% and 3.42%, respectively, indicating good long‐term NH_3_ sensing stability. Compared with the previously discussed thermoelectric voltage response, the difference in the absolute output voltage is mainly attributed to the device connection configuration. In the series configuration with four thermoelectric fibers, the output voltages of the individual fibers can be added together. In contrast, the stability test was conducted using ten thermoelectric fibers connected in parallel, for which the open‐circuit voltage generally does not increase significantly with the number of fibers. Therefore, the absolute voltage values obtained from different device configurations should not be directly compared.

Figure 7f–i presents the Joule heating characteristics of PANI/TeNWs composite fibers and the corresponding temperature distribution images recorded by an infrared thermal camera. The saturation temperature can be modulated by adjusting the applied voltage. As shown in Figure 7f,g, the saturation temperature of the device increases from 41.8 to 45.2°C with the applied voltage rising from 0.01 to 0.1 V. Figure 7h displays the reversible heating and cooling behavior of the fibers under cyclic switching between 0.01 and 0.1 V. Figure 7i shows nearly identical temperature profiles over five consecutive cycles at 0.01 V, verifying its excellent heating stability. Therefore, this device can be integrated into textiles as a wearable heater, guaranteeing human safety at moderate voltages.

## 3 Conclusions

A critical challenge in developing micrometer‐scale chemical sensors lies in reconciling multiple detection functions within a single microfiber while preserving high performance. Here, we report a continuous wet‐spinning approach to fabricate composite microfibers that integrate chemically responsive PANI with high‐Seebeck‐coefficient TeNWs. At an optimal TeNW loading of 60 wt.%, the resulting PANI/TeNWs fiber exhibits a *S* of 59.9 µV K^−1^ and a power factor of 9.2 µW m^−1^ K^−2^, enabling passive temperature sensing down to 1 K without external power. The same platform simultaneously delivers excellent chemical detection: a near‐Nernstian pH response of 59.25 mV pH^−1^ (*R^2^* = 0.99) across pH 4–11, and rapid ammonia sensing with a 0.96 s response time at 50 ppm. By realizing both passive thermoelectric transduction and active chemical sensing in a single microfiber, this work provides a material solution that bypasses the traditional trade‐off between energy autonomy and multimodal detection. The strategy opens new avenues for miniaturized sensors in precision medicine and environmental monitoring.

## 4 Experimental Section

### 4.1 Materials

Aniline (≥ 99.5%, Sinopharm Chemical Reagent Co., Ltd.), Ammonium Persulfate (APS, AR, ≥ 98%, Aladdin), Hydrochloric Acid (HCl, AR, 36.0∼38.0%, Sinopharm Chemical Reagent Co., Ltd.), Ammonium Hydroxide (NH_3_·H_2_O, AR, 25∼28%, Sinopharm Chemical Reagent Co., Ltd.), m‐Cresol(GC, ≥ 99%, Aladdin), Camphorsulfonic Acid (CSA, ≥ 99%, Aladdin), Tellurium Dioxide (TeO_2_, 99.99%, Aladdin), Potassium Hydroxide (KOH, AR, ≥ 85% Aladdin), Ethylene Glycol (EG, CP, ≥ 99.0%, Sinopharm Chemical Reagent Co., Ltd.), Polyvinylpyrrolidone (PVP, BASF, K‐30, Shanghai Meryer Biochemical Technology Co., Ltd.), Sodium bicarbonate (NaHCO_3_, Shanghai Yurui Biotechnology & Pharmaceutical Co., Ltd.), Omeprazole(Yuekang Pharmaceutical Group Co., Ltd.). The abbreviations used in this work and their corresponding physical units are listed in the Note S1.

### 4.2 Fabrication of TeNWs

TeNWs were synthesized using a hydrothermal method. During this process, tellurium dioxide (TeO_2_) reacted with potassium hydroxide (KOH) to form TeO_3_^2−^, which was then slowly reduced to Te by the mild‐reducing agent EG, resulting in the formation of TeNWs. 2.1 mmol of tellurium dioxide and 5.96 mmol of potassium hydroxide were dissolved in 60 mL of ethylene glycol, followed by mechanical stirring at 90 ℃ for 30 min. Then, 0.2 g of PVP was added and stirred until completely dissolved. The resulting mixture was transferred into a Teflon‐lined autoclave and heated for 24 h. After cooling to room temperature, the supernatant was discarded, and the obtained precipitate was centrifuged and washed 6 times. Finally, the product was dried under vacuum.

### 4.3 Fabrication of PANI

First, aniline was subjected to vacuum distillation to obtain a colorless and transparent aniline solution. 1 mL of aniline was added to a 1 m hydrochloric acid solution and stirred at room temperature for 1 h. Subsequently, ammonium persulfate was weighed and dissolved in another portion of 1 M hydrochloric acid solution at a molar ratio of aniline: ammonium persulfate = 1 : 0.9. The above ammonium persulfate solution was added dropwise to the aniline solution at a rate of 2 mL min^−1^ under ice bath conditions. After the dropwise addition was completed, the resulting mixed solution was reacted at 0 ℃ for 10 h. The reaction solution was washed with water and suction‐filtered until the filtrate became colorless and transparent. Then, the product was dedoped in a 1 m ammonia solution for 12 h, followed by further water washing and suction filtration. Finally, the obtained product was dried in a vacuum drying oven at 60 ℃ for 12 h to obtain a powder which was labeled as (PANIeb). The obtained powder was then doped with camphorsulfonic acid and denoted as (PANIes).

### 4.4 Fabrication of PANI/TeNWs Fibers

PANI/TeNWs composite fibers were prepared using a wet spinning method. PANI powder and CSA (PANI : CSA molar ratio of 2 : 1) were dispersed in m‐cresol at a concentration of 18.75 mg mL^−1^. The mixture was stirred at 60 ℃ for 24 h, after which the pre‐dispersed TeNWs were added and stirred for 1 h. The resulting mixture was centrifuged at 3000 rpm, transferred to a syringe, and uniformly extruded into ethanol precipitation at a flow rate of 0.2 mL min^−1^ using an infusion pump with a 23 G needle (inner diameter = 330 µm). After soaking for several min to remove m‐cresol, the PANI/TeNWs fibers were collected and air dried.

### 4.5 Characterization

The sample composition and structure were characterized by means of an X‐ray diffractometer (XRD; Rigaku, SmartLab), a field‐emission scanning electron microscope (FE‐SEM, Phenom Pharos G1, Phenom‐World) equipped with an energy‐dispersive X‐ray spectrometer (EDS), and a transmission electron microscope (TEM). Elemental composition and chemical bonding were analyzed using X‐ray photoelectron spectroscopy (XPS, Escalab 250Xi, Thermo Scientific) and Raman spectroscopy (LabRAM HR‐800). Nanostructure characterization was performed using grazing‐incidence wide‐angle X‐ray scattering (GIWAXS) at the BL02U2 beamline of the Shanghai Synchrotron Radiation Facility (SSRF). The Electrical conductivity (*σ*) was calculated according to Equation (9):

(9) $$\sigma =\frac{L}{RA}$$

where *L*, *R*, and *A* represent the fiber length, resistance, and cross‐sectional area, respectively. *R* was measured using a Fluke 15B+ multimeter after coating both ends of the fiber with a silver paste. The fiber diameter was measured using a Zeiss Axioscope 5 microscope. The Seebeck coefficient (*S*) was determined using a PTM‐3 instrument (Jiayitong Technology). The output performance of the thermoelectric generator was evaluated using a volt‐ampere test system comprising a multimeter (Fluke 15B+), voltmeter (Advantest R6871E), and series of fixed resistors. The temperature gradient was established using a constant‐temperature heating plate (Sail & Avionics SET1010). The sensing performance was assessed using a Keithley DMM6500.

### 4.6 Passive Temperature Sensing

Four fibers with a length of 3 cm were sequentially connected onto a polyimide (PI) substrate using silver paste as conductive wires to fabricate a sensor device. Both sides of the fibers were connected to silver wires using silver paste and fixed with tape to ensure stability, followed by final encapsulation with PI. The sensor was connected to a Keithley DMM6500 digital multimeter for synchronous real‑time data acquisition. The temperature‐sensing signal was recorded as voltage fluctuations.

### 4.7 PH Sensing

The pH sensitivity of the PANI/TeNWs fibers was evaluated in a three‐electrode system using an electrochemical workstation (CHI660E, Chenhua Instruments, Shanghai, China). A 3 cm PANI/TeNWs fiber served as the working electrode (WE), a platinum sheet as the counter electrode (CE), and a saturated calomel electrode (SCE) as the reference electrode (RE). Open‐circuit potential (OCP) measurements were performed in various buffer solutions: pH 4–6 (acetic acid and sodium acetate solution), pH 7–8 (sodium dihydrogen phosphate and sodium dihydrogen phosphate buffer), pH 9–10 (sodium bicarbonate and sodium carbonate buffer), and pH 11 (sodium hydroxide and sodium carbonate buffer). All buffer solutions were 0.1 m in concentration and were purchased from Sinopharm Chemical Reagent Co., Ltd. The PANI/TeNWs fibers were tested without additional device fabrication or tape encapsulation. Real‐sample testing was conducted using commercially available products: (1) Sodium bicarbonate solution: Two sodium bicarbonate tablets (0.3 g tablet^−1^, Shanghai Anding Bio‐Pharmaceutical Co., Ltd.) were dissolved in 50 mL of deionized water. (2) Omeprazole solution: Two omeprazole enteric‐coated capsules (20 mg capsule^−1^, Yuekang Pharmaceutical) were opened, and the contents were dissolved in 50 mL of deionized water.

### 4.8 Gas Sensing

Gas Sensing: First, the gas bag was purged with nitrogen (N_2_) to ensure a clean environment, and then the target gas was injected at a specific concentration. The sensor was integrated into the test environment and connected to a Keithley DMM6500 tester for the synchronous acquisition of real‐time dynamic response data. Owing to the thinness of the individual fibers, the sensor device was constructed by stacking 10 fibers to increase the contact area, with both ends connected by silver wires. After drawing 1 mL of gas from the gas bag using a syringe, it was rapidly blown toward the sensor center while maintaining a distance of ∼1 cm from the sensor surface. For gas testing, relative changes in resistivity (Δ*R*/*R*_0_) were characterized using the following Equation (10):

(10) $$\Delta R/R_{0}\left(\%\right)=\frac{R-R_{0}}{R_{0}}$$

Where *R*_0_ is the resistance in the gas‐free state, and *R* is the resistance in the presence of gas.

## Author Contributions

**Dongmei Xie** (Investigation: Lead; Methodology: Lead; Writing – original draft: Lead), **Mengran Chen** (Validation: Lead; Formal Analysis: Lead), **Xuefei Zhang** (Investigation: Supporting), **Yan Xu** (Investigation: Supporting), **Weiye Geng** (Investigation: Supporting), **Zhe Tang** (Investigation: Supporting), **Si‐ze Lou** (Investigation: Supporting), **Shun Wan** (Resource: Supporting), **Zhenguo Liu** (Resource: Equal), **Heng Liu** (Funding: Equal), **Peng‐an Zong** (Conceptualization:Lead,Writing – review & editing:Lead; Funding: Equal; Supervision: Equal).

## Conflicts of Interest

The authors declare no conflicts of interest.

## Supporting information




**Supporting File**: advs76311‐sup‐0001‐SuppMat.docx.

## Data Availability

The data that support the findings of this study are available from the corresponding author upon reasonable request.
