# Supplementary material for: Thermoelectric PANI/TeNWs Fiber Based Microsensor for Passive Temperature and Active Chemical Sensing
Source: Adv Sci (Weinh). 2026 Jun 29:e76311. Online ahead of print. doi: 10.1002/advs.76311 (PMC13337051; doi:10.1002/advs.76311)
Supplement: Supplementary file 1 — Supporting File: advs76311‐sup‐0001‐SuppMat.docx. [file ADVS-9999-e76311-s001.docx]

**Supplemental information**

**Thermoelectric PANI/TeNWs Fiber Based Microsensor for Passive Temperature and Active Chemical Sensing**

*Dongmei Xie^1,#^, Mengran Chen^1,#^, Xuefei Zhang^2^, Yan Xu^1^, Weiye Geng^1^, Zhe Tang^1^, Si-ze Lou^1^, Shun Wan^1^, Zhenguo Liu^3^, Heng Liu^1,4,*^, Peng-an Zong^1,*^*

^1^College of Materials Science and Engineering, Nanjing Tech University, Nanjing 210009, China.

^2^State Key Laboratory of New Ceramics and Fine Processing, School of Materials Science and Engineering, Tsinghua University, Beijing 100084, China.

^3^Key Laboratory of Flexible Electronics of Zhejiang Province, Ningbo Institute of Northwestern Polytechnical University, Ningbo 315103, China.

^4^Advanced Institute for Materials Research (WPI-AIMR), Tohoku University, Sendai 980-8577, Japan.

E-mail: heng.liu.e1@tohoku.ac.jp (Dr. Heng Liu); [pazong@njtech.edu.cn (Dr. Peng-an Zong)](mailto:georgepazong@gmail.com;).

**Contents**

[Note 1. Abbreviations used in this paper and their corresponding physical units. 1](#_Toc17142)

[Figure S1. Digital photographs of the PANI/TeNWs composite fiber. 2](#_Toc5681)

[Figure S2. XRD pattern of the as-synthesized TeNWs. 3](#_Toc5787)

[Figure S3. FTIR spectrum of PANI. 4](#_Toc13243)

[Figure S4. XPS survey spectrum of PANI/TeNWs composite fibers. 5](#_Toc6963)

[Figure S5. EDS elemental mapping of sulfur (S) element for the PANI/TeNWs composite fiber. 6](#_Toc6772)

[Figure S6. SEM images of TeNWs synthesized at different temperatures. 7](#_Toc25887)

[Figure S7. Statistical distribution of TeNWs length and diameter at different temperatures. 8](#_Toc12169)

[Figure S8. Photograph of the PANI/TeNWs composite fiber with 70 wt% TeNWs content. 9](#_Toc1537)

[Figure S9. Thermoelectric properties of PANI/60 wt% TeNWs composite fibers spun with different needle diameters. 10](#_Toc676)

[Figure S10. Reproducibility of the thermoelectric properties of PANI/TeNWs composite fibers. 11](#_Toc26886)

[Figure S11. Photograph of the PANI/TeNWs fiber-based device for temperature sensing tests. 12](#_Toc19375)

[Figure S12. Anti-interference performance of the PANI/TeNWs composite fiber sensor. 13](#_Toc8760)

[Figure S13. Radar chart comparing the overall performance of different pH sensors based on the parameters summarized in Table S1. 14](#_Toc9029)

[Figure S14. Schematic energy band diagrams of PANI and TeNWs before composite formation. 15](#_Toc20883)

[Figure S15. Photograph of the gas sensing test setup for PANI/TeNWs fiber devices. 16](#_Toc13305)

[Figure S16. Schematic diagram of gas sensing test. 17](#_Toc32707)

[Figure S17. Gas sensing stability of the PANI/TeNWs composite fiber sensor toward 200 ppm NH](#_Toc21160)_[3](#_Toc21160)_ [under different external conditions. 18](#_Toc21160)

[Figure S18. Simultaneous temperature and NH](#_Toc18851)_[3](#_Toc18851)_ [sensing responses of the sensor under different temperature gradients. 19](#_Toc18851)

[Figure S19. Environmental stability of the PANI/TeNWs composite fiber after exposure to ambient air. 20](#_Toc7779)

[Figure S20. Long-term sensing stability of the sensor. 21](#_Toc6400)

[Table S1. Comparison of electrochemical performance parameters of different pH sensors. 22](#_Toc3263)

[Table S2. Comparison of gas-sensing performance parameters of reported gas-sensing materials toward different target gases. 23](#_Toc23599)

[Reference 25](#_Toc11657)

**Note 1. Abbreviations used in this paper and their corresponding physical units.**

| NO. | Symbols/acronyms | Full names | Units |
| --- | --- | --- | --- |
| 1 | *σ* | Electrical conductivity | S cm^−1^ |
| 2 | *S* | Seebeck coefficient | μV K^−1^ |
| 3 | *PF* | Power factor | μW m^−1^ K^−2^ |
| 4 | U | Output voltage | mV |
| 5 | *q* | Scattering vector | Å^-1^ or nm^-1^ |
| 6 | E*_f_* | Fermi level | eV |
| 7 | E*_v_* | Valence band maximum |  |
| 8 | *R*_s_ | Series resistance |  |
| 9 | *R*_tr_ | Transfer resistance |  |
| 10 | *R*_rec_ | Recombination resistance |  |
| 11 | CSA | Camphorsulfonic acid |  |
| 12 | ppm | Parts per million | - |
| 13 | ΔR/R₀ | Change in resistance / resistance at initial state | - |
| 14 | TeNWs | Tellurium Nanowires | - |
| 15 | PANI | Polyaniline | - |
| 16 | TEG | Thermoelectric generator | - |
| 17 | PVP | Polyvinylpyrrolidone | - |
| 18 | RSD | Relative Standard Deviation | - |
| 19 | FT-IR | Fourier-transform infrared spectroscopy | - |
| 20 | SCE | Saturated Calomel Electrode | - |
| 21 | OCP | Open-circuit potential | - |
| 22 | LUMO | Highest Occupied Molecular Orbital | - |
| 23 | HOMO | Lowest Unoccupied Molecular Orbital | - |
| 24 | GIWAXS | Grazing Incidence Wide Angle X-Ray Scattering | - |
| 25 | XRD | X-ray diffractometer | - |
| 26 | XPS | X-ray photoelectron spectroscopy | - |
| 27 | SEM | Scanning electron microscope | - |
| 28 | EDS | Energy dispersive X-ray spectrometer | - |
| 29 | TEM | Transmission electron microscopy | - |
| 30 | EMFs | Electromagnetic field | - |
| 31 | *r*_b_ | Bending radius | mm |
| 32 | RE | Reference electrode | - |
| 33 | CE | Counter electrode | - |
| 34 | WE | Working electrode | - |
| 35 | MFC | Mass Flow Controller | - |


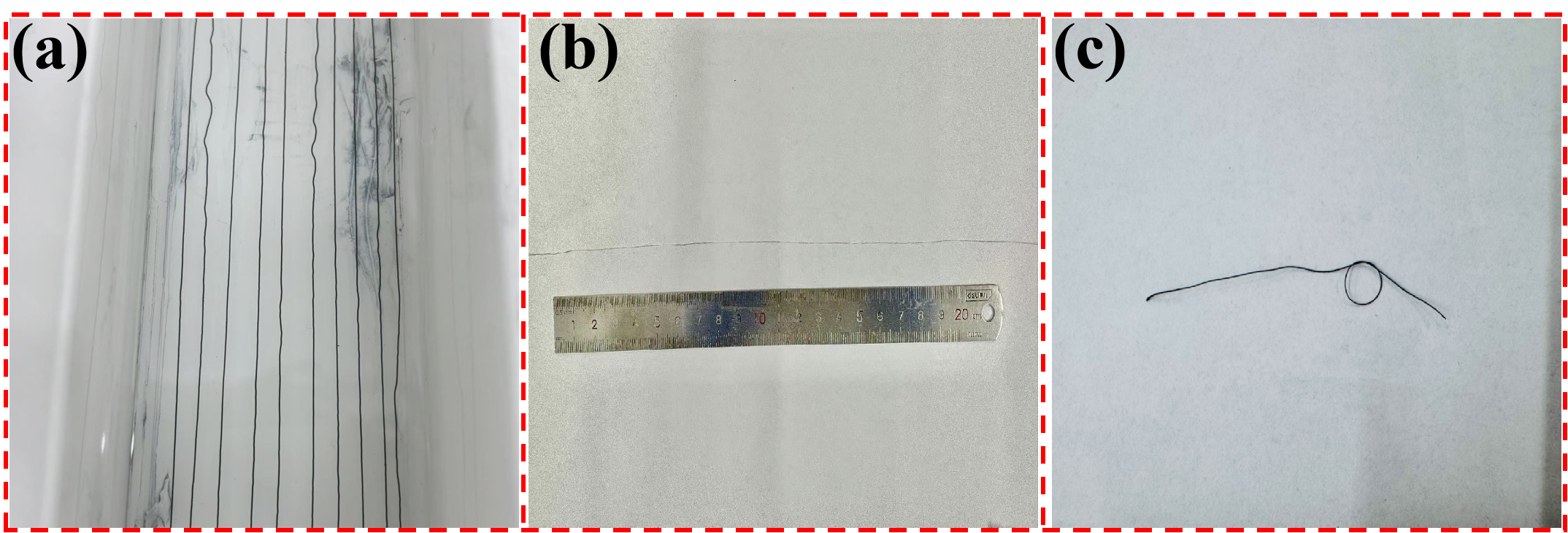


**Figure S1. Digital photographs of the PANI/TeNWs composite fiber.**

(a) As-dried composite fiber. (b) Single composite fiber. (c) Knotted composite fiber, demonstrating its flexibility.


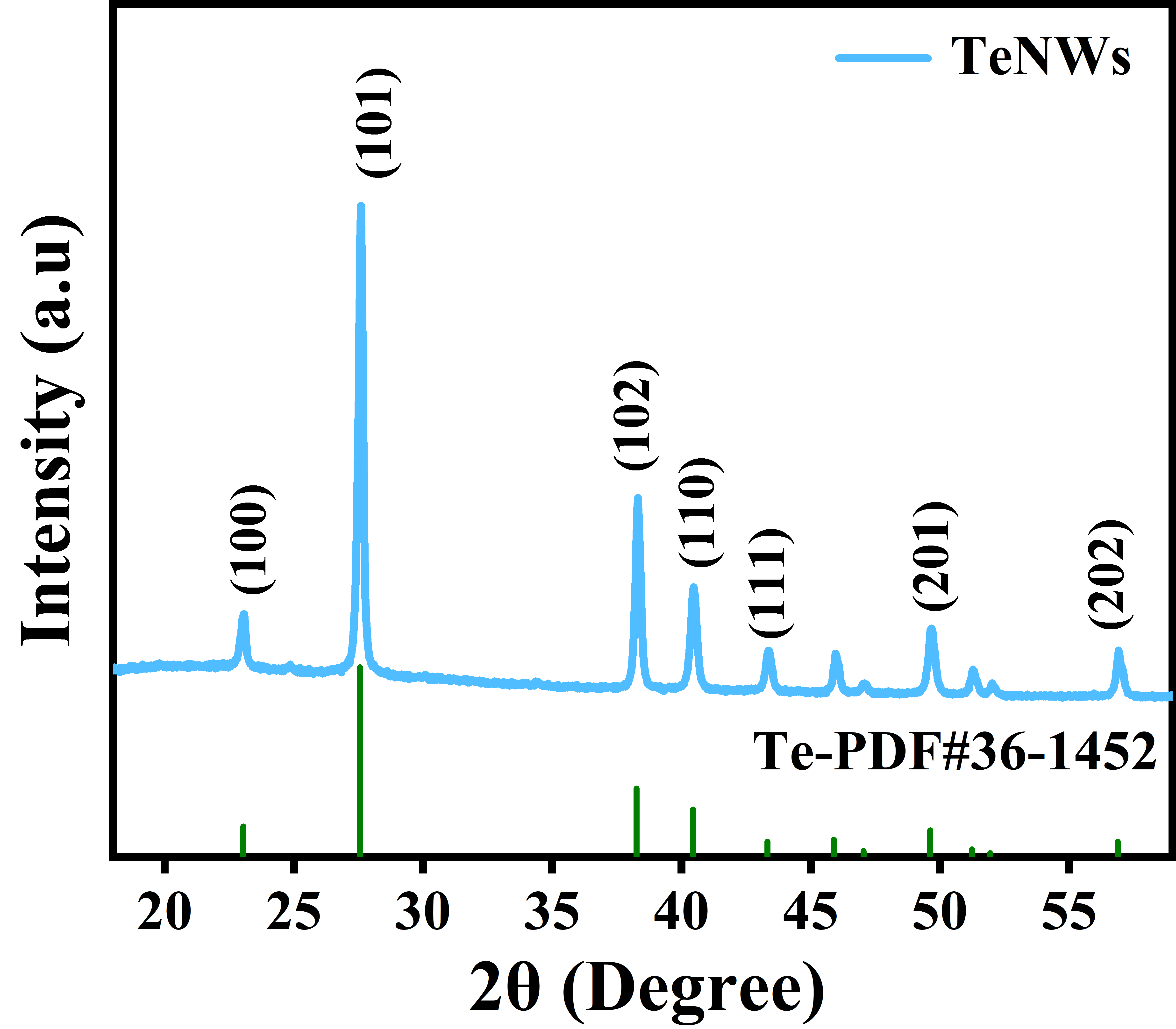


**Figure S2. XRD pattern of the as-synthesized TeNWs.**

All diffraction peaks are well indexed to the standard pattern of hexagonal tellurium (JCPDS#36-1452), confirming the successful synthesis of TeNWs.


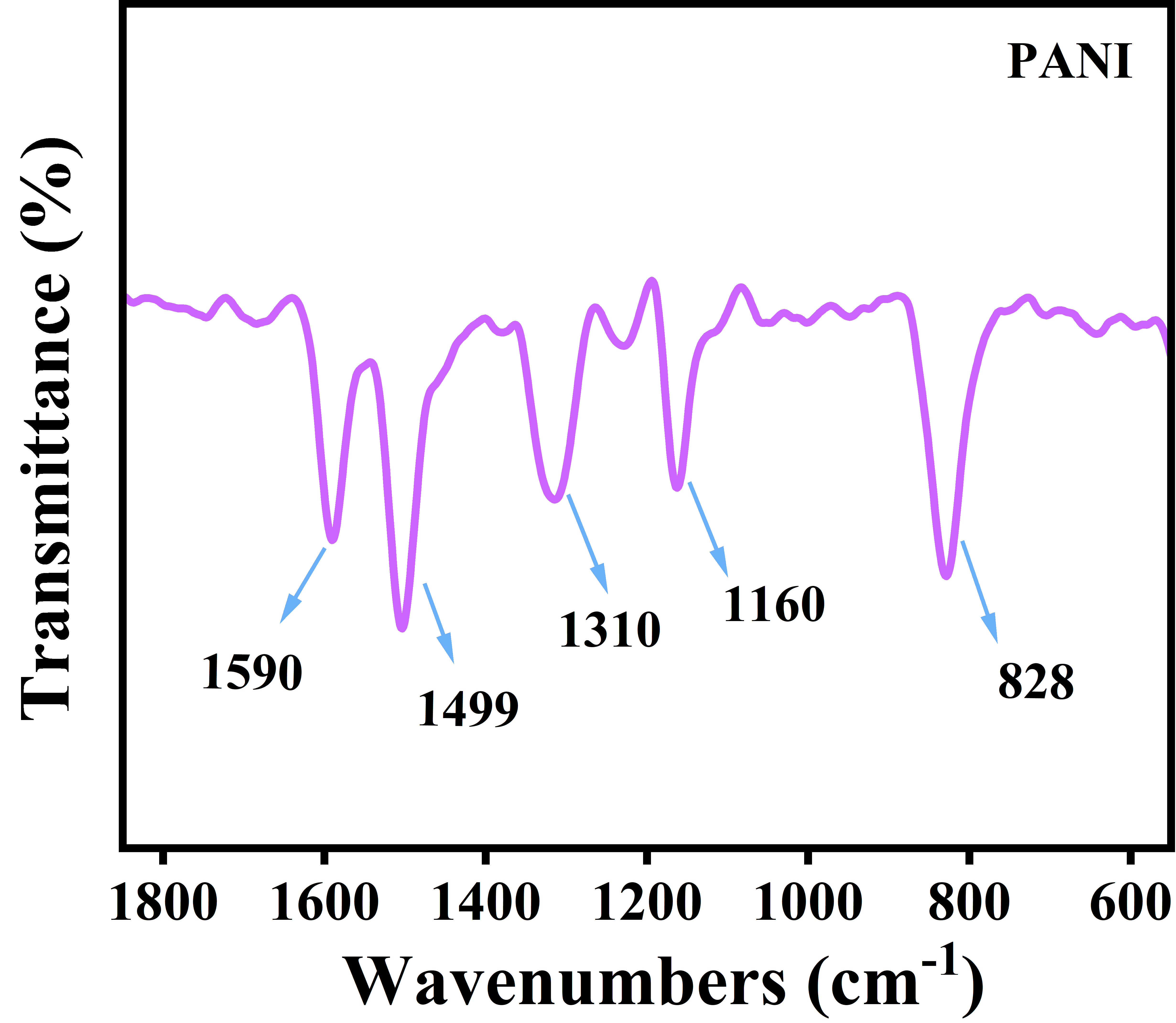


**Figure S3. FTIR spectrum of PANI.**

The characteristic peaks at 828, 1160, 1310, 1499, and 1590 cm^−1^ correspond to C–H bending, the conducting form of PANI (electron delocalization), C–N stretching, and C=C stretching of the benzenoid and quinoid rings, respectively, confirming the emeraldine base structure of the synthesized PANI.


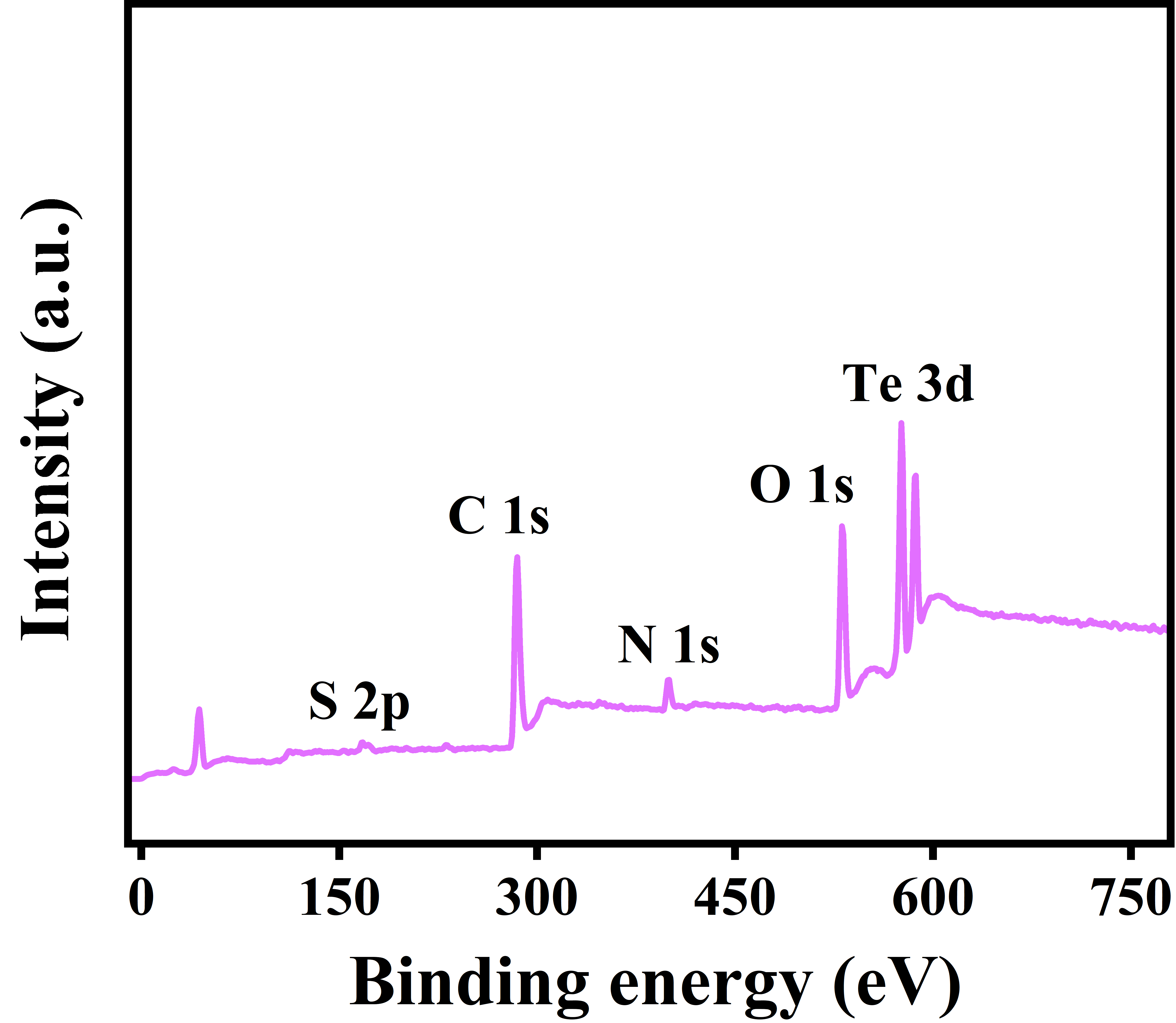


**Figure S4. XPS survey spectrum of PANI/TeNWs composite fibers.**

The spectrum confirms the presence of C, N, O, S, and Te elements, with a flat background indicating good data quality.


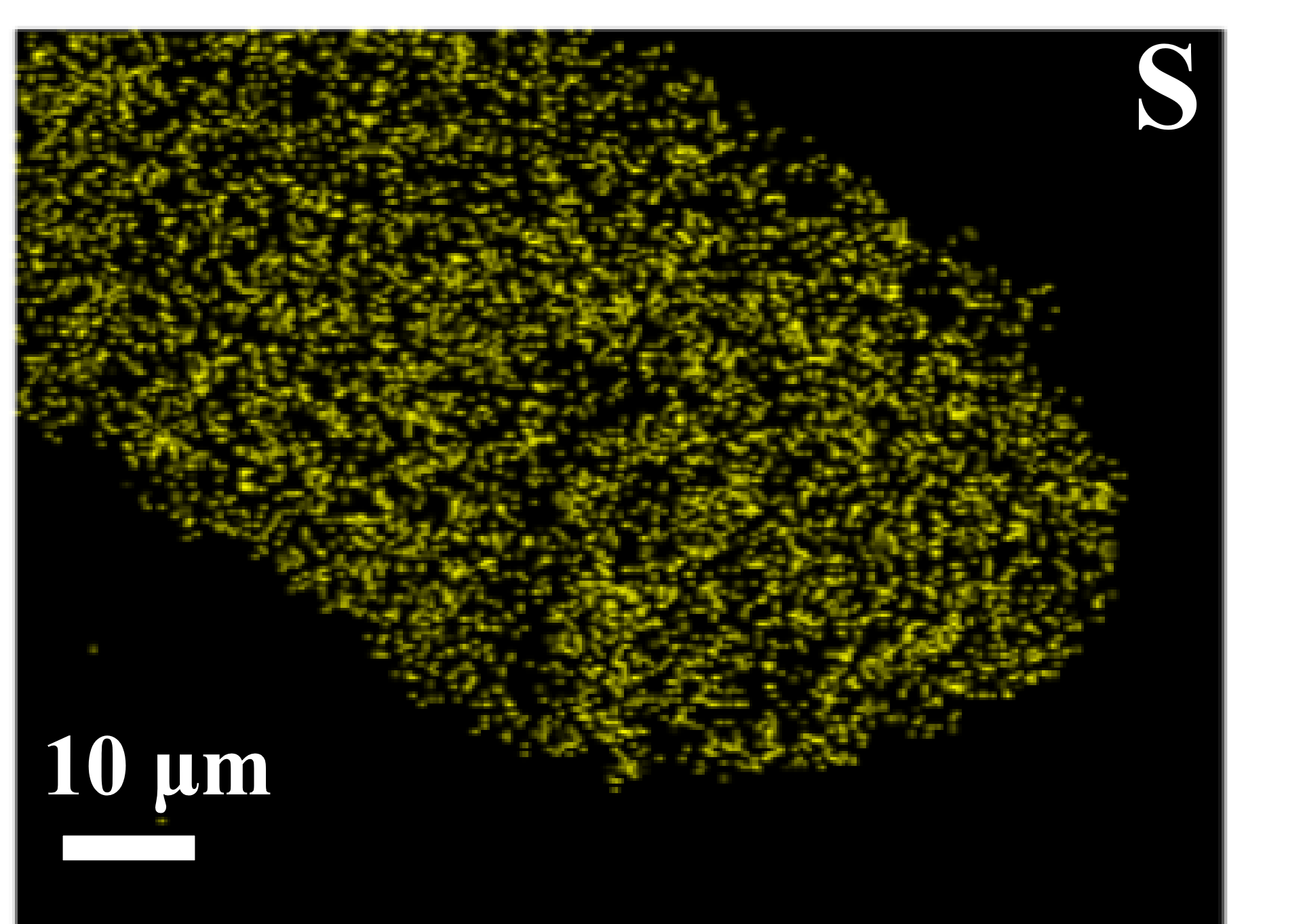


**Figure S5. EDS elemental mapping of sulfur (S) element for the PANI/TeNWs composite fiber.**

The uniform distribution of S (originating from the camphorsulfonic acid dopant) throughout the fiber cross-section confirms the homogeneous doping of PANI within the composite.


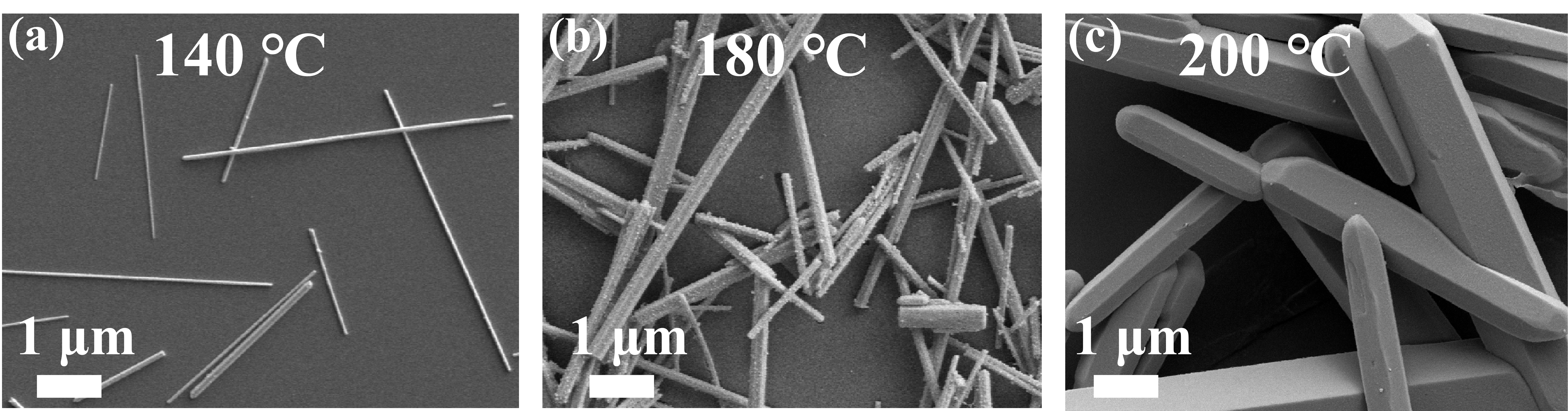


**Figure S6. SEM images of TeNWs synthesized at different temperatures.**

(a) 140 ℃, (b) 180 ℃, and (c) 200℃. The morphology evolves from short nanorods at 140 ℃ to uniform nanowires with high aspect ratio at 160 ℃ (see Figure 3a in the main text), and then to shorter, thicker structures at higher temperatures, demonstrating the critical role of synthesis temperature in controlling TeNWs morphology.


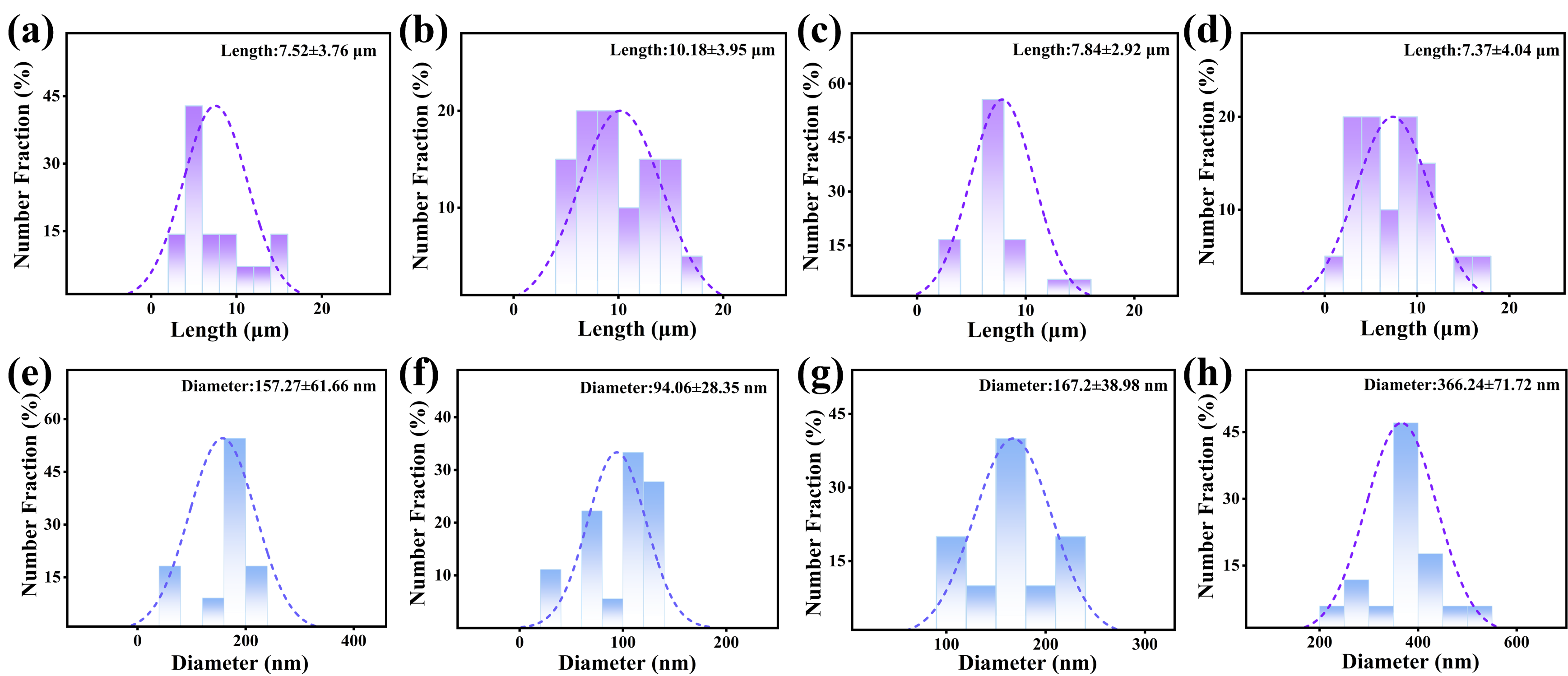


**Figure S7. Statistical distribution of TeNWs length and diameter at different temperatures.**

(a-d) Length distribution at (a) 140 ℃, (b) 160 ℃, (c) 180 ℃ and (d) 200 ℃. (e-h) Corresponding diameter distribution histograms at (e) 140 ℃, (f) 160 ℃, (g) 180 ℃ and (h) 200 ℃. The TeNWs synthesized at 160 ℃ exhibit the most uniform length distribution and the highest aspect ratio, consistent with the optimal thermoelectric performance shown in Figure 3c,d.


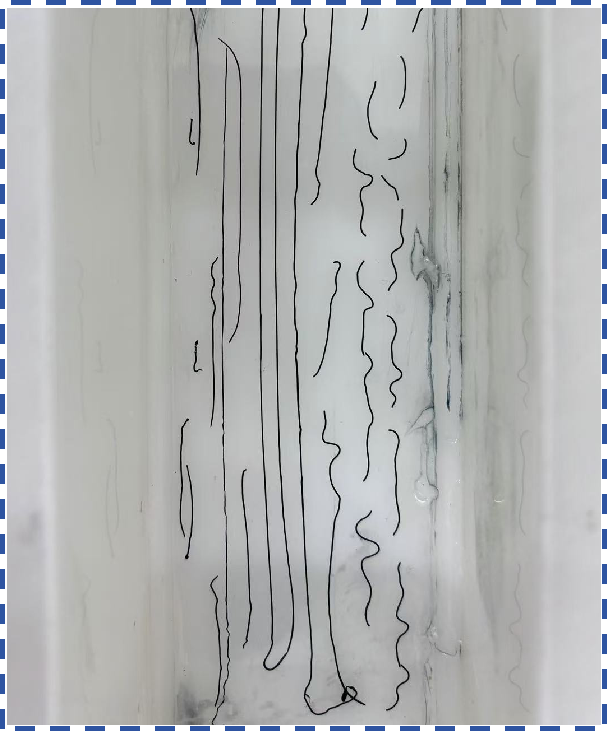


**Figure S8. Photograph of the PANI/TeNWs composite fiber with 70 wt% TeNWs content.**

The fiber exhibits discontinuous segments and poor spinnability, indicating that excessive TeNWs loading (>70 wt%) disrupts the continuous fiber formation during wet spinning. This observation supports the selection of 60 wt% TeNWs as the optimal composition for further studies.


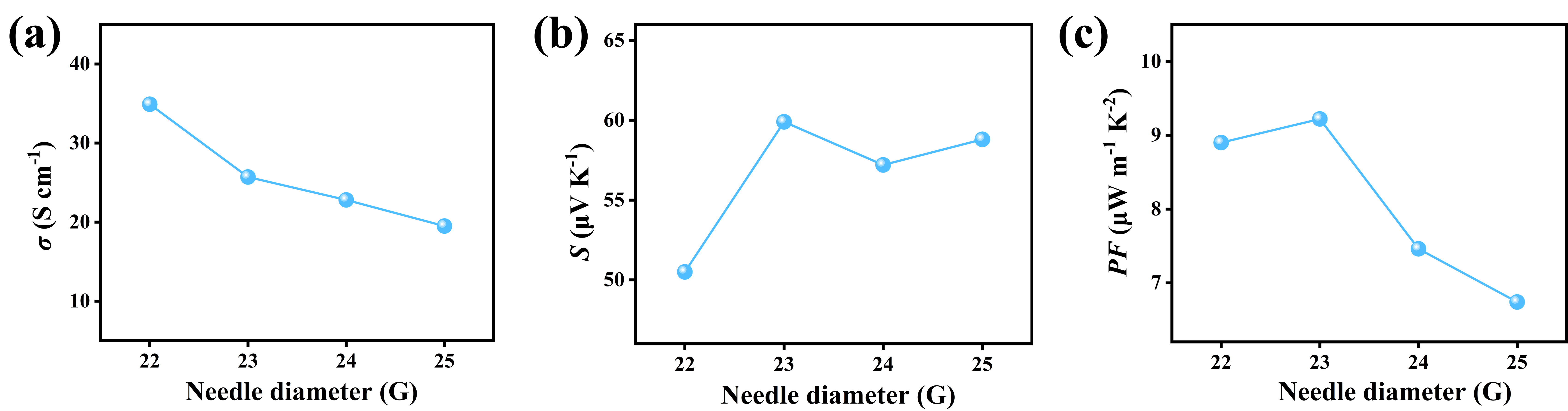


**Figure S9. Thermoelectric properties of PANI/60 wt% TeNWs composite fibers spun with different needle diameters.**

(a) Electrical conductivity, *σ*, (b) Seebeck coefficient *S,* and (c) Power factor, *PF* for fibers fabricated using 22G, 23G, and 25G needles. The 23G needle yields the highest power factor, attributed to the optimal balance between fiber orientation and structural uniformity during wet spinning.


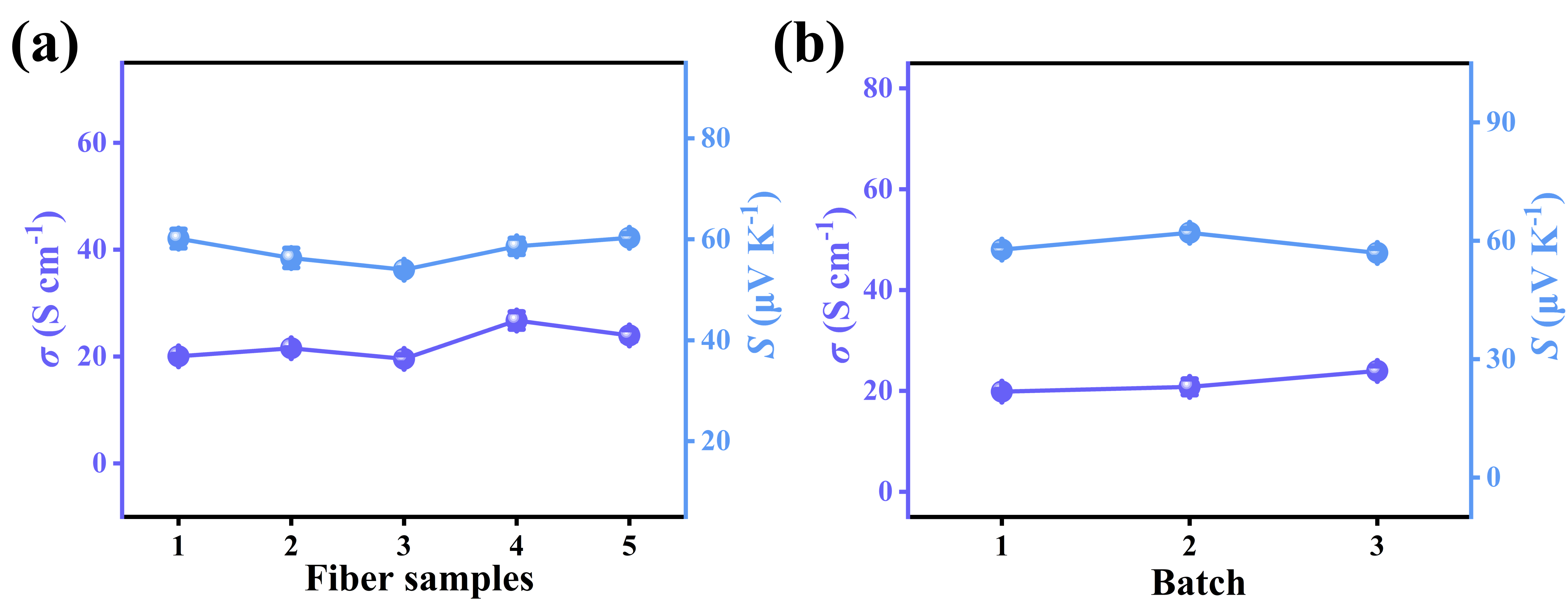


**Figure S10. Reproducibility of the thermoelectric properties of PANI/TeNWs composite fibers.**

(a) sample-to-sample variations in electrical conductivity (*σ*) and Seebeck coefficient (*S*) among five individual fiber samples, and (b) batch-to-batch variations in *σ* and *S* among three independently prepared batches. The five individual fiber samples yield average *σ* = 22.3 ± 3.0 S cm^−1^ and *S* = (57.9 ± 2.7 μV K^−1^, corresponding to relative standard deviations (RSD) of 13.5% and 4.7%, respectively, indicating good sample-to-sample uniformity. In addition, the batch-to-batch comparison among three independently prepared batches shows consistent thermoelectric properties, with average *σ* and *S* values of 21.5 ± 2.1 S cm^−1^ and 58.9 ± 2.7 μV K^−1^, respectively. The small error bars confirm the good fabrication reproducibility of the wet‑spun composite fibers.


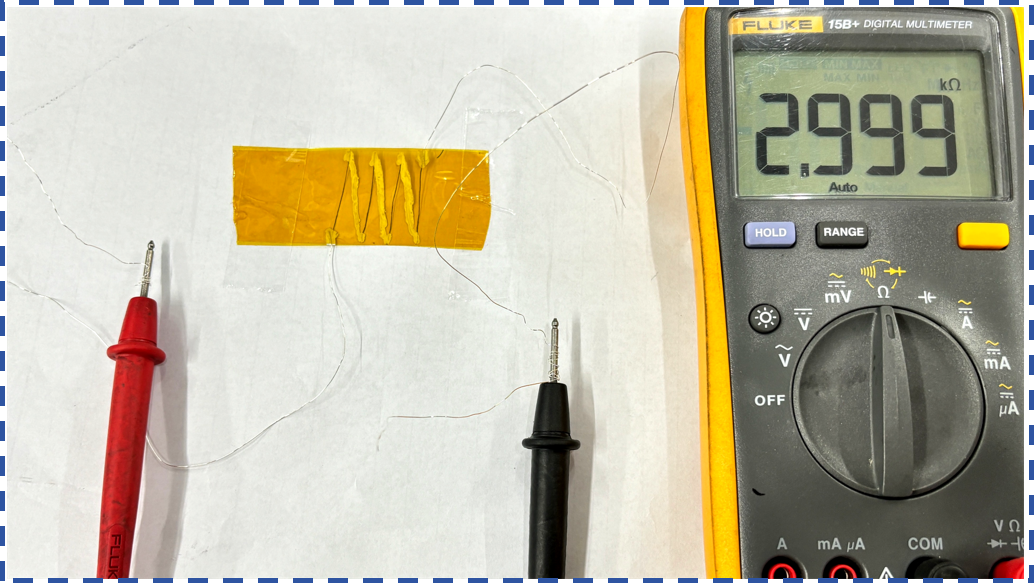


**Figure S11. Photograph of the PANI/TeNWs fiber-based device for temperature sensing tests**.

The device consists of four strands of PANI/60 wt% TeNWs composite fibers (each 3 cm in length) connected in series using silver paste and silver wires, then encapsulated with polyimide (PI) tape. This configuration corresponds to the device structure illustrated in Figure 4a and was used for the passive temperature sensing measurements presented in Figures 4c-i.


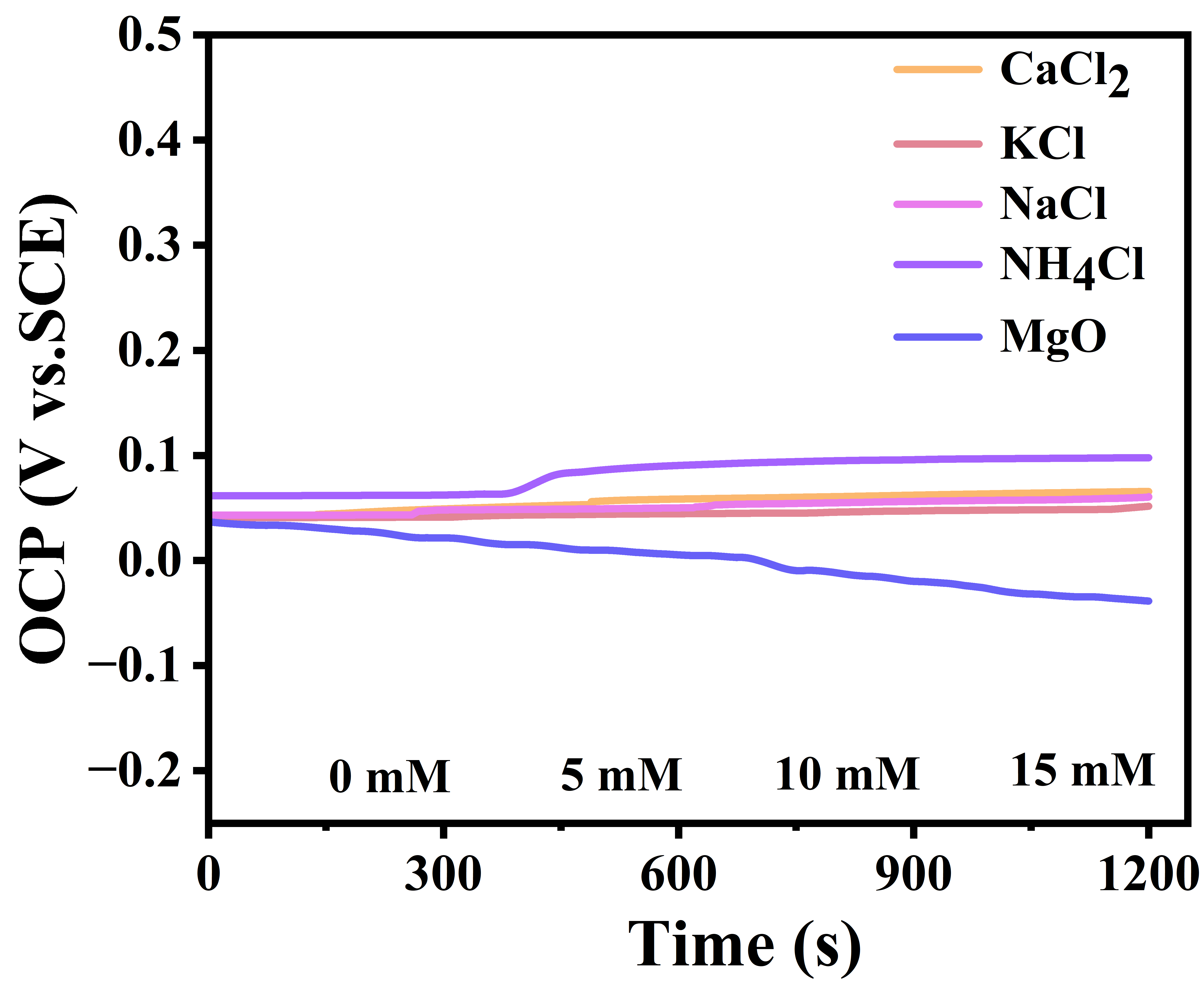


**Figure S12. Anti-interference performance of the PANI/TeNWs composite fiber sensor.**

Open-circuit potential (OCP) response upon sequential addition of potential interfering species at concentrations ranging from 0 to 15 mM. For common cations (NH_4_^+^, Ca^2+^, Na^+^, K^+^; added as chloride salts), the OCP exhibits minimal variation with increasing concentration, demonstrating excellent selectivity of the sensor toward H^+^ over other physiologically relevant ions. In a separate experiment with MgO addition, a significant potential shift is observed at 15 mM, attributed to the formation of Mg(OH)_2_ which increases local OH^−^ concentration and alters solution pH, rather than direct ionic interference. This behavior further confirms that the sensor responds primarily to H^+^ activity.


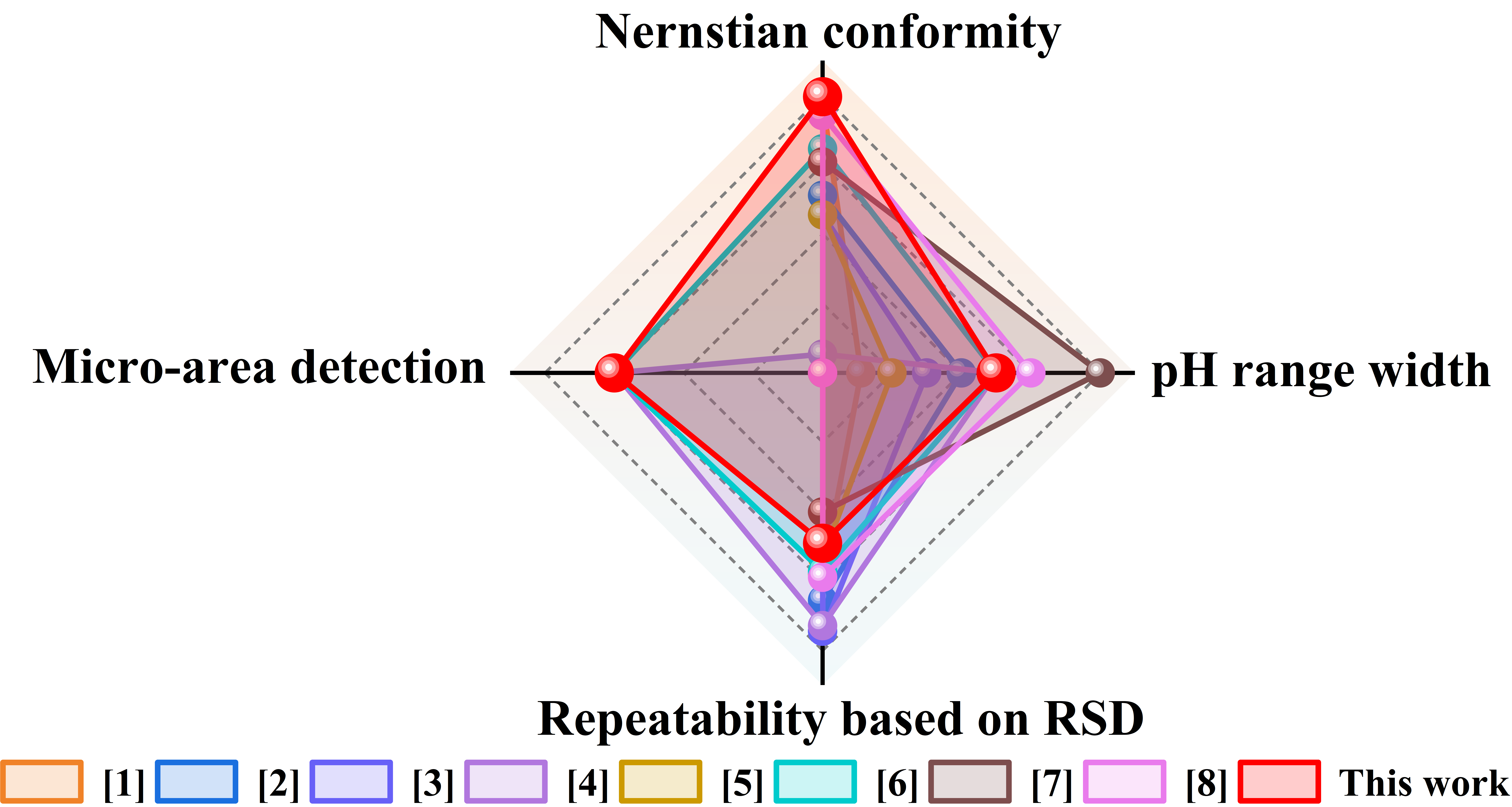


**Figure S13. Radar chart comparing the overall performance of different pH sensors based on the parameters summarized in Table S1.**

Nernstian conformity was calculated as:

$$Nernstian conformity = 1－\frac{\left| S_{i}－S_{N} \right|}{S_{N}}$$

where S_i_ is the sensitivity and S_N_ is the theoretical Nernstian response of 59.16 mV pH^−1^ at room temperature. The pH range width was calculated as (pH_max_－pH_min_). For repeatability, the RSD values were reversely normalized because a lower RSD indicates better repeatability. For micro-area detection capability, sensors with and without this capability were assigned values of 1 and 0, respectively.


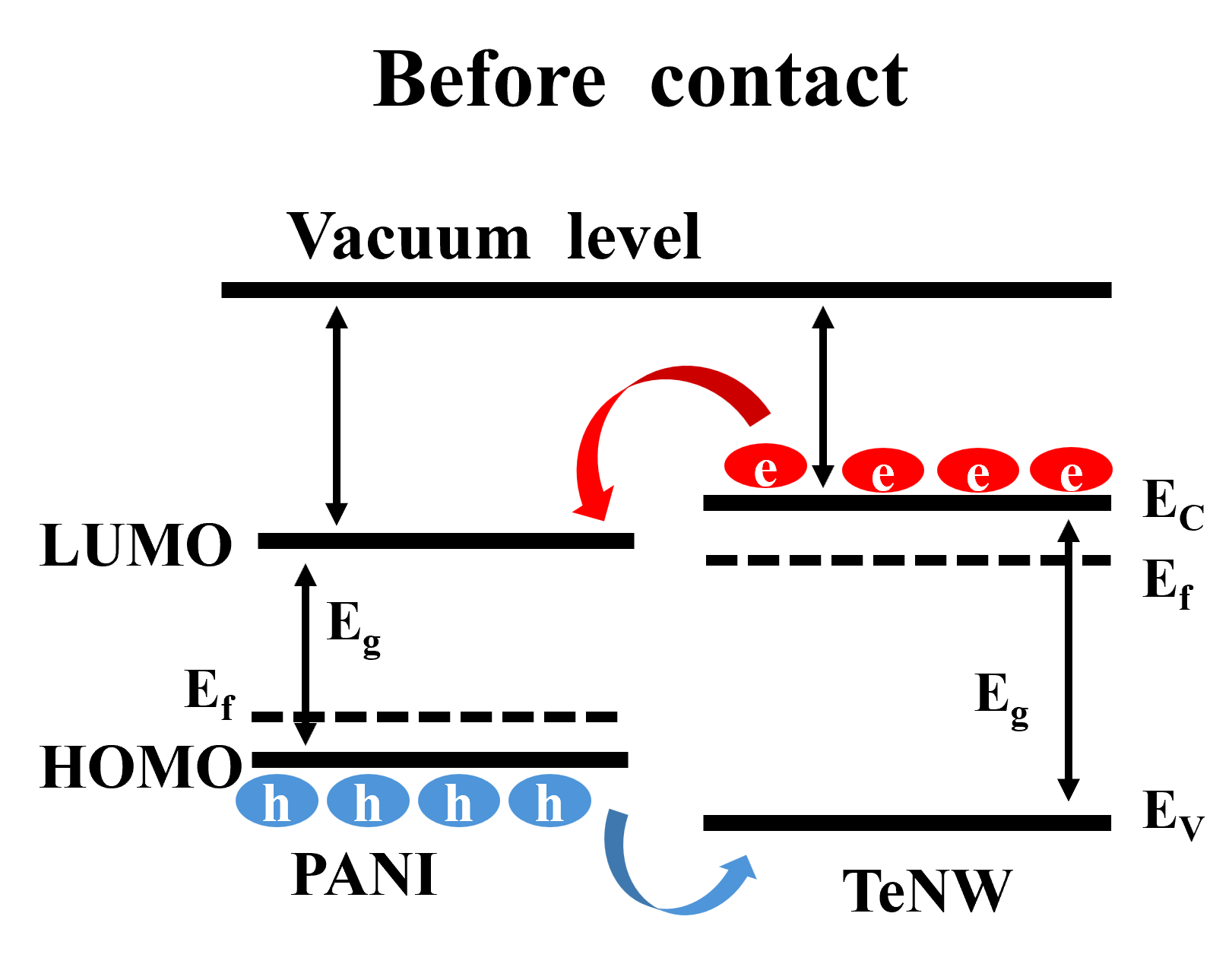


**Figure S14. Schematic energy band diagrams of PANI and TeNWs before composite formation.**

Both PANI and TeNWs are p-type semiconductors. Prior to contact, the Fermi level of TeNWs is lower than that of PANI. Upon interfacial contact, holes diffuse from PANI (higher Fermi level) to TeNWs (lower Fermi level), leading to the formation of a depletion region at the heterojunction as illustrated in Figure 6a. This band alignment underpins the charge transfer behavior and the gas sensing mechanism discussed in the main text.


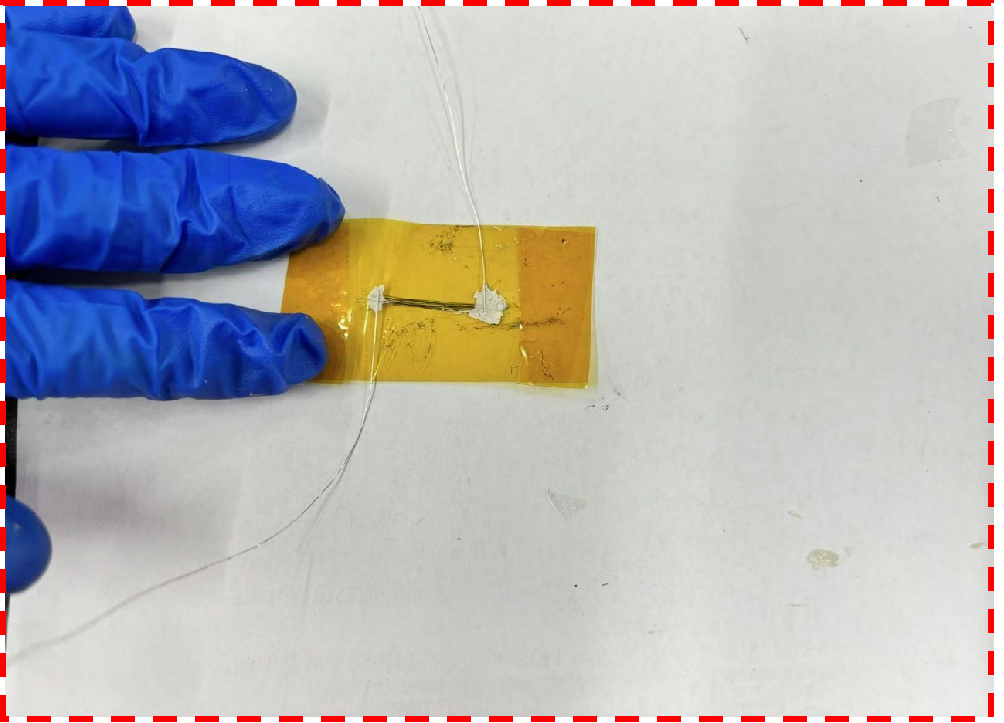


**Figure S15. Photograph of the gas sensing test setup for PANI/TeNWs fiber devices**.

The sensor device was fabricated by stacking ten strands of PANI/60 wt% TeNWs composite fibers (each 3 cm in length) to increase the contact area. Silver wires were connected to both ends of the fiber bundle using silver paste to ensure reliable electrical contact. This device configuration corresponds to the gas sensing measurements presented in Figures 6d-i, where the sensor was connected to a Keithley DMM6500 digital multimeter for real-time resistance monitoring upon exposure to target gases.

**
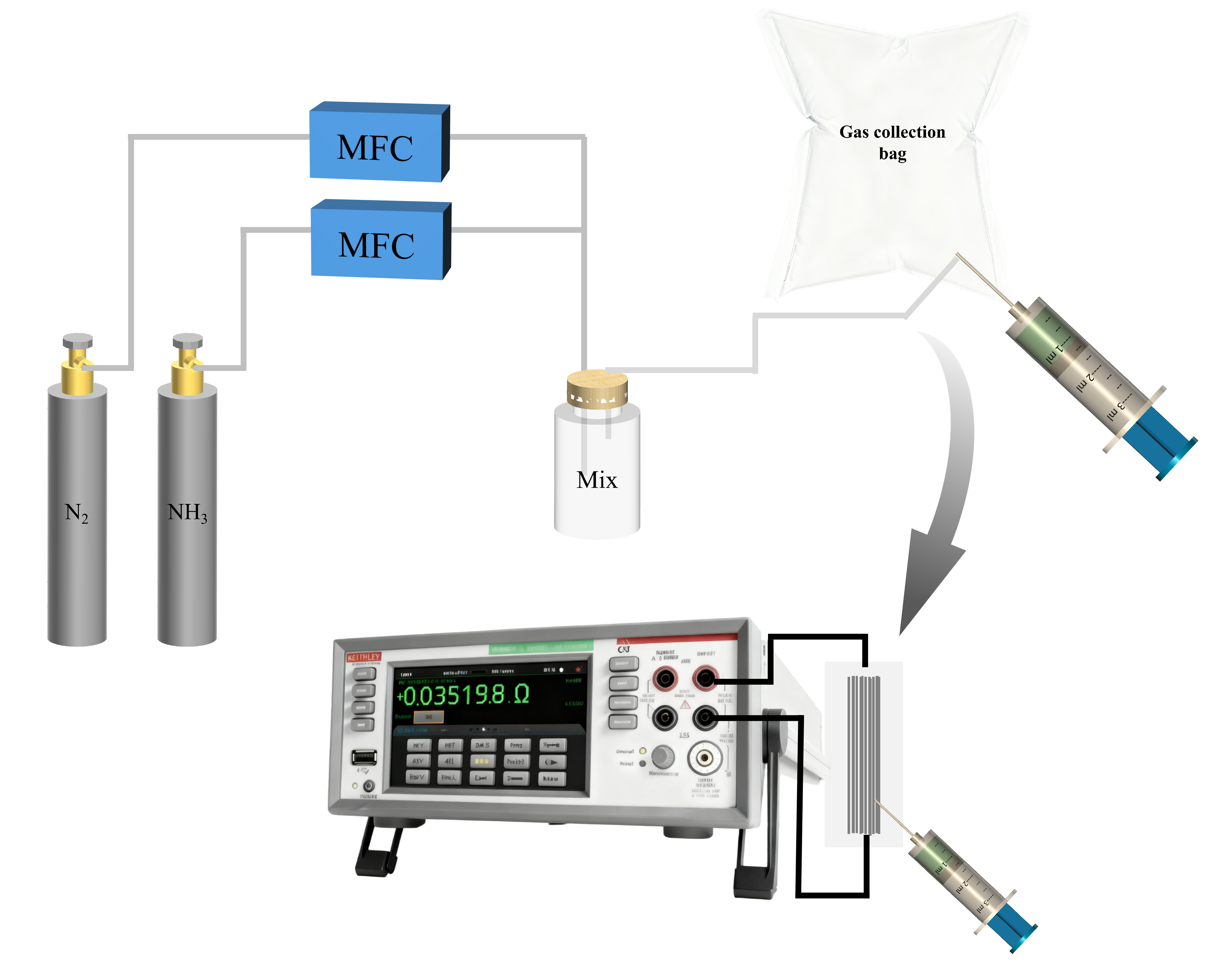
**

**Figure S16. Schematic diagram of gas sensing test.**

The gas sensing test setup is illustrated above. The procedure involves the following steps:

1).Clean the gas bag multiple times with nitrogen to remove residual gases.

2).Use a gas flow meter to control the concentration of the gas mixture.

3).Release the mixed gas for 5 minutes to ensure uniform concentration before sampling.

4).Collect 50 μL of the gas mixture at different concentrations using a micro-sampler.

5).Gently blow the gas towards the center of the sensor, maintaining a distance of approximately 1 cm between the needle tip and the sensor.

6).Connect the sensor to the Keithley DMM6500 digital multimeter using silver wires for measurement.

This method ensures precise and reproducible gas sensing measurements, enabling accurate evaluation of the sensor performance.

**
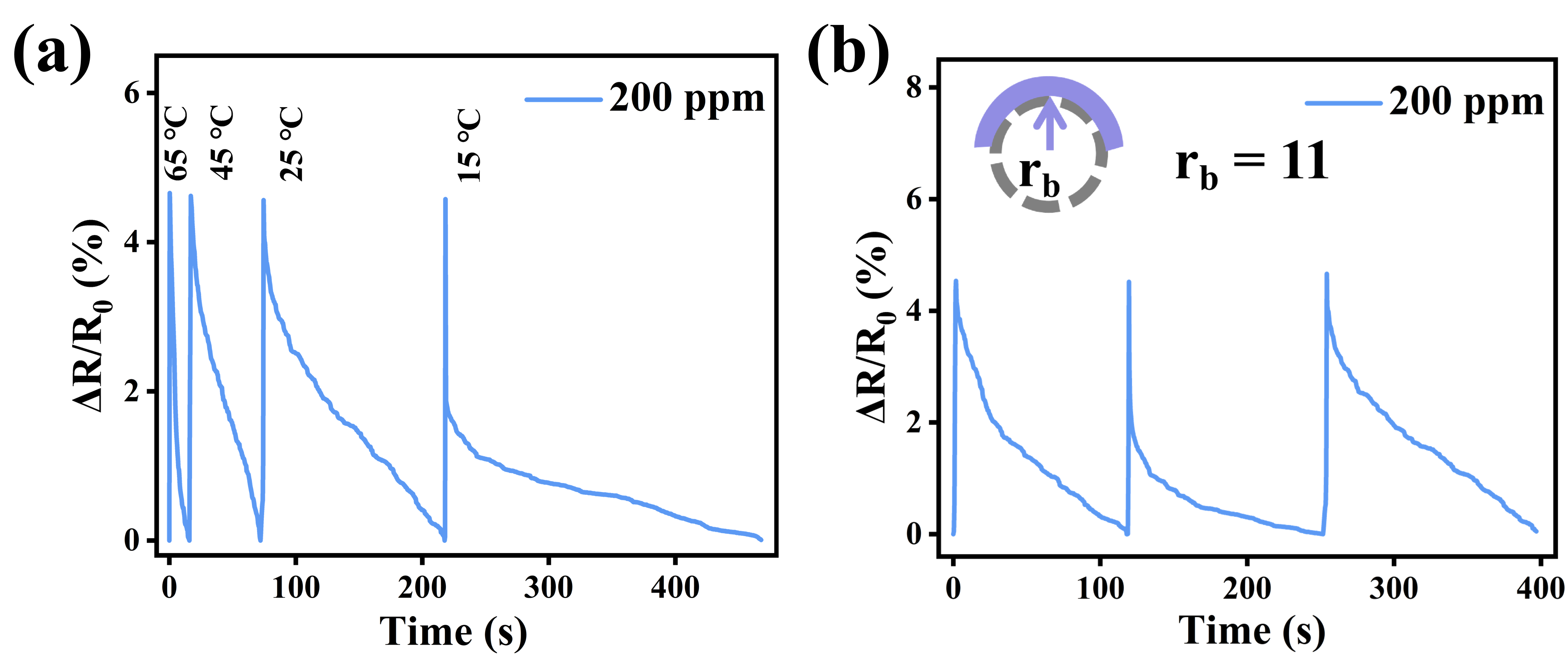
**

**Figure S17. Gas sensing stability of the PANI/TeNWs composite fiber sensor toward 200 ppm NH_3_ under different external conditions.**

(a) different temperatures of 15, 25, 45, and 65 ℃, and (b) repeated bending deformation. The sensor maintains a clear response to 200 ppm NH_3_ at different temperature, indicating stable NH_3_ sensing capabilities under different thermal environments. In addition, the sensor exhibits a repeatable response after bending , suggesting that the PANI/TeNWs composite fiber maintains continuous conductive pathways. These results further demonstrate its mechanical reliability and its potential for flexible NH_3_ sensing applications.

**
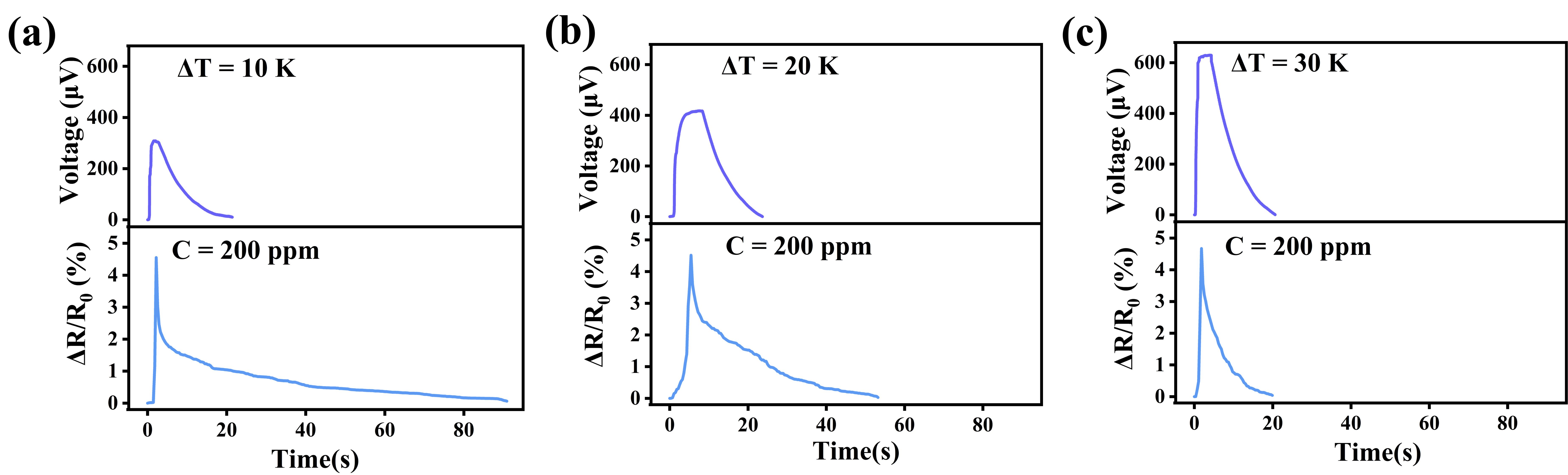
**

**Figure S18. Simultaneous temperature and NH_3_ sensing responses of the sensor under different temperature gradients.**

The upper panels show the thermoelectric voltage responses at (a) ΔT = 10 K, (b) ΔT = 20 K, and (c) ΔT = 30 K, while the lower panels show the corresponding relative resistance changes during exposure to 200 ppm NH_3_.

**
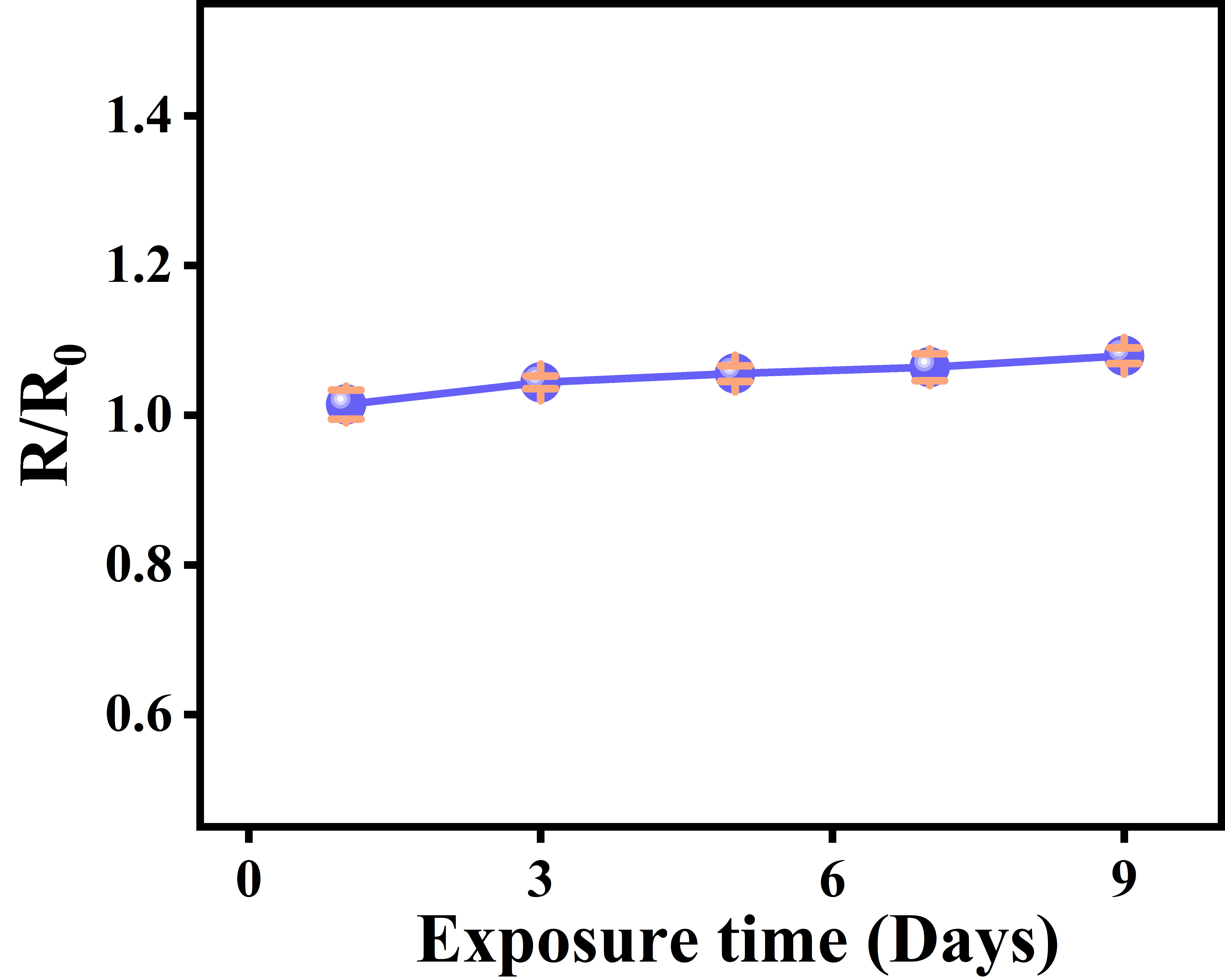
**

**Figure S19. Environmental stability of the PANI/TeNWs composite fiber after exposure to ambient air.**

The normalized resistance (*R*/*R*_0_) remains close to 1.0 with only a slight increase over 9 days, indicating that the composite fiber maintains a stable conductive network and good environmental stability after air exposure.

**Figure S20. Long-term sensing stability of the sensor.**

(a) Thermoelectric voltage response of the sensor under ΔT = 10 K after 30 days of storage. (b) OCP output stability of the sensor in a pH = 4 buffer solution after 30 days of storage. (c) Relative resistance responses of the sensor to 200 ppm NH_3_ after 1, 9, and 30 days of storage. It should be noted that the stability test was performed using a parallel configuration of ten thermoelectric fibers, whereas the thermoelectric output measurement presented above was conducted using a series configuration of four thermoelectric fibers.

**Table S1. Comparison of electrochemical performance parameters of different pH sensors.**

| **Sensing material** | **pH range** | **Sensitivity (mV pH**^−1^**)** | **RSD (%)** | **Micro-area Detection** | **Ref.** |
| --- | --- | --- | --- | --- | --- |
| PANI membrane | 5.5-8.6 | 58.57 | 2.4 | No | [^[[1]](#endnote-0)^] |
| PANI@OPU/PP spunbonded nonwoven fabric | 2.0-8.0 | 67.67 | 1.5 | No | [^[[2]](#endnote-1)^] |
| Flexible 3D porous PANI matrix | 4.0-9.0 | 69.33 | 0.4 | No | [^[[3]](#endnote-2)^] |
| Inkjet-printed PANI/PPy polymer sensing layer | 3.0−10.0 | 81.20 | 0.6 | Yes | [^[[4]](#endnote-3)^] |
| Acid-doped 3D PANI nanofiber network | 5.0-9.0 | 69.31 | 3.0 | No | [^[[5]](#endnote-4)^] |
| Microneedle membrane pH electrode | 5.0-8.5 | 54.60 | 2.5 | Yes | [^[[6]](#endnote-5)^] |
| PEDOT:PSS/PANI composite-coated sonogel–carbon electrode | 2.0–12.0 | 53.50 | 4.6 | No | [^[[7]](#endnote-6)^] |
| PANI nanowire-modified carbon fiber cloth electrode | 4.0-12.0 | 60.90 | 2.3 | No | [^[[8]](#endnote-7)^] |
| Wet-spun PANI/TeNWs composite fiber | 4.0-11.0 | 59.25 | 3.5 | Yes | This work |

**Table Footnote:** RSD: relative standard deviation. Micro-area detection capability was determined based on whether the sensing device had a microneedle, fiber, or probe geometry that enables localized pH detection in confined or irregular microenvironments; planar films or electrodes with relatively large sensing areas were classified as "No" unless localized detection was demonstrated.

**Table S2. Comparison of gas-sensing performance parameters of reported gas-sensing materials toward different target gases.**

| **Material** | **Range**  **(ppm)** | **C (ppm)** | **Response (%)** | **Response/recovery time** | **Selectivity** | **Stability (days,response variation)** | **Operating Temperature (℃)** | **Linearity** | **Ref.** |
| --- | --- | --- | --- | --- | --- | --- | --- | --- | --- |
| PANI/rGO-NH_2_ | 25-125 | 50 | 79.8 | 14/66 | NH_3_ | 26, <0.1% | RT | 0.994 | [^[[9]](#endnote-8)^] |
| PANI/MWCNTs | 5-1000 | 5 | 1.4 | 0.81/200 | NH_3_ | - | RT | 0.980 | [^[[10]](#endnote-9)^] |
| PANI/Ti_3_C_2_T_x_ | 1-1000 | 50 | 167 | 17/70 | NH_3_ | 15, 48.5% | RT | 0.990 | [^[[11]](#endnote-10)^] |
| Au/PANI/WS_2_ | 5-500 | 100 | 286.1 | 24/26 | NH_3_ | 193, 3.7% | RT | 0.993 | [^[[12]](#endnote-11)^] |
| PANI/LIG | 30-150 | 60 | 68 | 53/167 | NH_3_ | 31, ~0.3% | RT | 0.877 | [^[[13]](#endnote-12)^] |
| rGO/MoS_2_-LIG | 0.2-5 | 1 | 0.5 | 360/720 | NO_2_ | - | 60 | 0.995 | [^[[14]](#endnote-13)^] |
| Needlelike LIG | 0.5-2.5 | 1 | ~0.6 | 134/388 | NO | - | RT | 0.992 | [^[[15]](#endnote-14)^] |
| Ag/LIG | 0.5-2.5 | 1 | 1.2 | 40/291 | NO_2_ | - | RT | 0.990 | [^[[16]](#endnote-15)^] |
| VO_x_-doped LIG | 0.3-0.7 | 1 | 2.5 | 217/650 | NO_2_ | 16, ~4% | RT | 0.997 | [^[[17]](#endnote-16)^] |
| In_2_O_3_-ZnO/LIG | 200-1000 | 500 | 27.48 | 48/169 | CH_4_ | 29, ~1% | RT | 0.994 | [^[[18]](#endnote-17)^] |
| ZnO/LIG | 0.2-1 | 1 | 24 | 21/23 | C_3_H_6_O | - | RT | 0.992 | [^[[19]](#endnote-18)^] |
| WO_3_/LIG | 0.02-0.1 | 0.02 | 24 | 32/78 | H_2_S | 210, <5% | RT | 0.992 | [^[[20]](#endnote-19)^] |
| WS_2_@PANI | 5-500 | 100 | 216.3 | 25/39 | NH_3_ | 30, ~20% | RT | 0.97 | [^[[21]](#endnote-20)^] |
| WS_2_/MoO_3_ | 0.2-50 | 3 | 31.58 | 57/226 | NH_3_ | 30, ~30% | RT | 0.990 | [^[[22]](#endnote-21)^] |
| MXene/GO/CuO/ZnO | 25-200 | 100 | 59.9 | 26/25 | NH_3_ | 45, ~3% | RT | 0.998 | [^[[23]](#endnote-22)^] |
| PANI/GO | 30-230 | 70 | 9.6 | 51/28 | NH_3_ | 50, ~16% | RT | 0.997 | [^[[24]](#endnote-23)^] |
| **Material** | **Range**  **(ppm)** | **C (ppm)** | **Response (%)** | **Response/recovery time** | **Selectivity** | **Stability (days,response variation)** | **Operating Temperature (℃)** | **Linearity** | **Ref.** |
| PANI/Nb_2_CT*_x_* | 10-50 | 10 | 205.4 | 371/352 | NH_3_ | - | RT | 0.962 | [^[[25]](#endnote-24)^] |
| WS_2_/CuO | 1-50 | 5 | 40.5 | 100/805 | NH_3_ | 42, ~5% | RT | 0.994 | [^[[26]](#endnote-25)^] |
| S-rGO/WS_2_ | 10-50 | 10 | 250 | 100/480 | NH_3_ | - | 28 | 0.983 | [^[[27]](#endnote-26)^] |
| WS_2_/WO_3_ | 1-100 | 100 | 450.2 | 202/191 | NH_3_ | - | 125 | - | [^[[28]](#endnote-27)^] |
| PANI/TeNWs | 50-500 | 50 | 2.4 | 0.96/255 | NH_3_ | 30, 3.42% | RT | 0.985 | This work |

**Note:** The values in the stability column represent the storage duration in air and the corresponding variation in sensing response relative to the initial response.

**Abbreviations:** rGO-NH_2_: Amino-functionalized reduced graphene oxide; WS_2_: Tungsten disulfide; LIG: Laser-induced graphene; VO_x_: Vanadium oxide; MoO_3_: Molybdenum trioxide; GO: Graphene oxide; S-rGO: Sulfur-doped reduced graphene oxide; WO_3_: Tungsten trioxide.

**Reference**

1. [] Li, Y.; Mao, Y.; Xiao, C.; et al. Flexible pH Sensor Based on a Conductive PANI Membrane for pH Monitoring. *RSC Adv.* **2020**, 10, 21-28. https://doi.org/10.1039/C9RA09188B. [↑](#endnote-ref-0)
2. [] Zhu X, Sun H, Yu B, et al. A Flexible pH Sensor Based On Polyaniline@Oily Polyurethane/Polypropylene Spunbonded Nonwoven Fabric. *RSC Adv*. **2024**, 14, 5627-5637. https://doi.org/10.1039/D3RA07878G. [↑](#endnote-ref-1)
3. [] Zhao, Y.; Yu, Y.; Zhao, S.; et al. Highly Sensitive pH Sensor Based on Flexible Polyaniline Matrix for Synchronal Sweat Monitoring. *Microchem. J.* **2023**, 185, 108092. https://doi.org/10.1016/j.microc.2022.108092. [↑](#endnote-ref-2)
4. [] Zea, M.; Texidó, R.; Villa, R.; et al. Specially Designed Polyaniline/Polypyrrole Ink for a Fully Printed Highly Sensitive pH Microsensor. *ACS Appl. Mater. Interfaces*. **2021**, 13, 33524-33535. https://doi.org/10.1021/acsami.1c08043. [↑](#endnote-ref-3)
5. [] Bai, Y.; Zhu, R.; Zhao, J.; et al. Super-Nernstian Model Based on Acid-Doped Polyaniline pH Sensor. *Microchem. J*. **2024**, 203, 110715. https://doi.org/10.1016/j.microc.2024.110715. [↑](#endnote-ref-4)
6. [] García-Guzmán JJ, Pérez-Ràfols C, Cuartero M, Crespo GA. Toward In Vivo Transdermal pH Sensing With a Validated Microneedle Membrane Electrode. *ACS Sens.* **2021**, 6, 1129-1137. https://doi.org/10.1021/acssensors.0c02397. [↑](#endnote-ref-5)
7. [] Sainz-Calvo, Á. J.; Sierra-Padilla, A.; Bellido-Milla, D.; et al. Fast, Economic, and Improved Nanostructured Polymeric pH Sensor for Agrifood Analysis. *Chemosensors*. **2025**, 13, 63. <https://doi.org/10.3390/chemosensors13020063>. [↑](#endnote-ref-6)
8. [] Hossain, M. S.; Padmanathan, N.; Badal, M. M. R.; et al. Highly Sensitive Potentiometric pH Sensor Based on Polyaniline-Modified Carbon Fiber Cloth for Food and Pharmaceutical Applications. *ACS Omega*. **2024**, 9, 40122-40133. https://doi.org/10.1021/acsomega.4c06090. [↑](#endnote-ref-7)
9. [] Kalaleh, H. A.; Masri, K.; Allaf, A. W.; Alhabbal M. O., Mahmoud M. Polyaniline/Reduced Graphene Oxide-NH_2_ Hybrid for Selective NH_3_ Sensing. *Mater. Chem. Phys.* **2026**, 359, 132558. https://doi.org/10.1016/j.matchemphys.2026.132558. [↑](#endnote-ref-8)
10. [] Chen, M.; Liu, H.; Zhang, X.; et al. Interfacial Molecular Ordering of PANI/MWCNT for Hazard and Vital Biophysical Signal Monitoring. *Adv. Funct. Mater.* **2026**, 36, e22905. https://doi.org/10.1002/adfm.202522905. [↑](#endnote-ref-9)
11. [] Pan, Y.; Li, X.; Shi, H.; et al. High-Performance Flexible NH_3_ Sensor Based on PANI/Ti_3_C_2_Tx Composites for Real-Time Fish Spoilage Detection. *Chem. Eng. J.* **2025**, 526, 170817. https://doi.org/10.1016/j.cej.2025.170817. [↑](#endnote-ref-10)
12. [] Wang, P.; Tang, C.; Zhang, L.; Lu, Y.; Huang, F. Hierarchical 0D/1D/2D Au/PANI/WS_2_ Ternary Nanocomposite NH_3_ Sensor with High Performance and Fast Response/Recovery for Food Spoilage Detection. *Chem. Eng. J.* **2024**, 496, 153998. https://doi.org/10.1016/j.cej.2024.153998. [↑](#endnote-ref-11)
13. [] Yang, L.; Mao, L.; Du, S.; et al. Highly Sensitive and Fast Response/Recovery Ammonia Sensor Based on PANI/LIG at Room Temperature. *Sens. Actuators B Chem.* **2025**, 437, 137710. https://doi.org/10.1016/j.snb.2025.137710. [↑](#endnote-ref-12)
14. [] Yang, L.; Yi, N.; Zhu, J.; et al. Novel Gas Sensing Platform Based on a Stretchable Laser-Induced Graphene Pattern with Self-Heating Capabilities. *J. Mater. Chem. A.* **2020**, 8, 6487-6500. https://doi.org/10.1039/C9TA07855J. [↑](#endnote-ref-13)
15. [] Yang, L.; Zheng, G.; Cao, Y.; et al. Moisture-Resistant, Stretchable NO_x_ Gas Sensors Based on Laser-Induced Graphene for Environmental Monitoring and Breath Analysis. *Microsyst.* *Nanoeng*. **2022**, 8, 78. https://doi.org/10.1038/s41378-022-00414-x. [↑](#endnote-ref-14)
16. [] Yang, L.; Ji, H.; Meng, C.; et al. Intrinsically Breathable and Flexible NO_2_ Gas Sensors Produced by Laser Direct Writing of Self-Assembled Block Copolymers. *ACS Appl. Mater. Interfaces*. **2022**, 14, 17818-17825. https://doi.org/10.1021/acsami.2c02061. [↑](#endnote-ref-15)
17. [] Yang, L.; Yan, J.; Meng, C.; et al. Vanadium Oxide-Doped Laser-Induced Graphene Multi-Parameter Sensor to Decouple Soil Nitrogen Loss and Temperature. *Adv. Mater.* **2023**, 35, 2210322. https://doi.org/10.1002/adma.202210322. [↑](#endnote-ref-16)
18. [] Yang, L.; Fu, W.; Mao, L.; et al. In_2_O_3_-ZnO/Laser-Induced Graphene Nanocomposites for a Highly Sensitive, Room-Temperature, Flexible Methane Gas Sensor. *ACS Appl. Nano Mater.* **2025**, 8, 7510-7519. https://doi.org/10.1021/acsanm.4c07323. [↑](#endnote-ref-17)
19. [] Yang, L.; Fu, W.; Wang, Y.; et al. ZnO/LIG Nanocomposites to Detect Acetone Gas at Room Temperature with High Sensitivity and Low Detection Limit. *Chem. Eng. J.* **2025**, 519, 164857. https://doi.org/10.1016/j.cej.2025.164857. [↑](#endnote-ref-18)
20. [] Yang, L.; Mao, L.; Wang, Z.; et al. Highly Sensitive, Room-Temperature Hydrogen Sulfide Gas Sensor Based on Tungsten Trioxide/Laser-Induced Graphene. *Chem. Eng. J.* **2025**, 521, 166393. https://doi.org/10.1016/j.cej.2025.166393. [↑](#endnote-ref-19)
21. [] Wang, P.; Tang, C.; Song, H.; Zhang, L.; Lu, Y.; Huang, F. 1D/2D Heterostructured WS_2_@PANI Composite for Highly Sensitive, Flexible, and Room Temperature Ammonia Gas Sensor. *ACS Appl. Mater. Interfaces.* **2024**, 16, 14082-14092. https://doi.org/10.1021/acsami.4c01136. [↑](#endnote-ref-20)
22. [] Ou, Y.; Niu, W.; Zhou, Y.; Guo, Y.; Gao, C.; Wang, Y. Mesoporous WS_2_/MoO_3_ Hybrids for High-Performance Trace Ammonia Detection. *ACS Appl. Mater. Interfaces.* **2022**, 14, 39062-39071. https://doi.org/10.1021/acsami.2c10773. [↑](#endnote-ref-21)
23. [] Seekaew, Y.; Kamlue, S.; Wongchoosuk, C. Room-Temperature Ammonia Gas Sensor Based on Ti_3_C_2_T_x_ MXene/Graphene Oxide/CuO/ZnO Nanocomposite. *ACS Appl. Nano Mater.* **2023**, 6, 9008-9020. https://doi.org/10.1021/acsanm.3c01637. [↑](#endnote-ref-22)
24. [] Mohammed, H. Y.; Farea, M. A.; Sayyad, P. W.; et al. Selective and Sensitive Chemiresistive Sensors Based on Polyaniline/Graphene Oxide Nanocomposite: A Cost-Effective Approach. *J. Sci.: Adv. Mater. Devices.* **2022**, 7, 100391. https://doi.org/10.1016/j.jsamd.2021.08.004. [↑](#endnote-ref-23)
25. [] Wang, S.; Jiang, Y.; Liu, B.; et al. Ultrathin Nb_2_CT_x_ Nanosheets-Supported Polyaniline Nanocomposite: Enabling Ultrasensitive NH_3_ Detection. *Sens. Actuators B Chem.* **2021**, 343, 130069. https://doi.org/10.1016/j.snb.2021.130069. [↑](#endnote-ref-24)
26. [] Luo, H.; Shi, J.; Liu, C.; et al. Design of p-p Heterojunctions Based on CuO Decorated WS_2_ Nanosheets for Sensitive NH_3_ Gas Sensing at Room Temperature. *Nanotechnology.* **2021**, 32, 445502. https://doi.org/10.1088/1361-6528/ac1800. [↑](#endnote-ref-25)
27. [] Wang, X.; Huang, B.; Wu, X.; Gu, D.; Li, X. Enhanced Ammonia Sensing Properties of rGO/WS_2_ Heterojunction Based Chemiresistive Sensor by Marginal Sulfonate Decoration. *Sens. Actuators B Chem.* **2021**, 337, 129776. https://doi.org/10.1016/j.snb.2021.129776. [↑](#endnote-ref-26)
28. [] Zheng, Y.; Sun, L.; Liu, W.; Wang, C.; Dai, Z.; Ma, F. Tungsten Oxysulfide Nanosheets for Highly Sensitive and Selective NH_3_ Sensing. *J. Mater. Chem. C.* **2020**, 8, 4206-4214. https://doi.org/10.1039/C9TC06686A. [↑](#endnote-ref-27)
